# Characterization and validation of a preventative therapy for hypertrophic cardiomyopathy in a murine model of the disease

**DOI:** 10.1073/pnas.2002976117

**Published:** 2020-08-28

**Authors:** Helena M. Viola, Ashay A. Shah, Victoria P. A. Johnstone, Henrietta Cserne Szappanos, Mark P. Hodson, Livia C. Hool

**Affiliations:** ^a^School of Human Sciences (Physiology), The University of Western Australia, Crawley, WA 6009, Australia;; ^b^Victor Chang Innovation Centre, Victor Chang Cardiac Research Institute, Sydney, NSW 2010, Australia;; ^c^School of Pharmacy, University of Queensland, Woolloongabba, QLD 4072, Australia

**Keywords:** hypertrophic cardiomypathy, L-type calcium channel, mitochondria, therapy

## Abstract

Currently there is an unmet need for treatments that can prevent hypertrophic cardiomyopathy (HCM). Using a murine model we previously identified that HCM causing cardiac troponin I mutation Gly203Ser (*cTnI-G203S*) is associated with increased mitochondrial metabolic activity, consistent with the human condition. These alterations precede development of the cardiomyopathy. Here we examine the efficacy of in vivo treatment of *cTnI-G203S* mice with a peptide derived against the α-interaction domain of the cardiac L-type calcium channel (AID-TAT) on restoring mitochondrial metabolic activity, and preventing HCM. *cTnI-G203S* or age-matched *wt* mice were treated with active or inactive AID-TAT. Following treatment, targeted metabolomics was utilized to evaluate myocardial substrate metabolism. Cardiac myocyte mitochondrial metabolic activity was assessed as alterations in mitochondrial membrane potential and flavoprotein oxidation. Cardiac morphology and function were examined using echocardiography. Cardiac uptake was assessed using an in vivo multispectral imaging system. We identified alterations in six biochemical intermediates in *cTnI-G203S* hearts consistent with increased anaplerosis. We also reveal that AID-TAT treatment of precardiomyopathic *cTnI-G203S* mice, but not mice with established cardiomyopathy, restored cardiac myocyte mitochondrial membrane potential and flavoprotein oxidation, and prevented myocardial hypertrophy. Importantly, AID-TAT was rapidly targeted to the heart, and not retained by the liver or kidneys. Overall, we identify biomarkers of HCM resulting from the cTnI mutation Gly203Ser, and present a safe, preventative therapy for associated cardiomyopathy. Utilizing AID-TAT to modulate cardiac metabolic activity may be beneficial in preventing HCM in “at risk” patients with identified Gly203Ser gene mutations.

## Significance

Hypertrophic cardiomyopathy affects 1:500 of the general population. Current drug therapy is used to manage symptoms in patients. There is an unmet need for treatments that can prevent the cardiomyopathy. Here we identify biomarkers of hypertrophic cardiomyopathy resulting from causing cardiac troponin I mutation Gly203Ser, and present a safe, nontoxic, preventative approach for the treatment of associated cardiomyopathy.

Hypertrophic cardiomyopathy (HCM) is a primary myocardial disorder that affects 1:500 of the general population (1). It is the leading cause of sudden cardiac death in the young (5- to 15-y-olds) (2). Drug therapy is used to manage symptoms in patients with overt HCM (3), but no treatment exists that can reverse or prevent the cardiomyopathy. Therefore, determining strategies to prevent the development of HCM is critical for effective treatment of the disease.

Genetic mutations in sarcomeric proteins are associated with the development of HCM. Cardiac troponin (cTn) is a sarcomeric protein complex that consists of three subunits (cTnT, cTnI, and cTnC) and plays a critical role in regulating cardiac contraction and relaxation. The entire cTn complex is anchored to tropomyosin via TnT. TnI regulates contraction in response to changes in intracellular calcium (4). During the relaxed state, TnI inhibits actin–myosin interaction. When calcium binds to TnC, TnI undergoes a conformational change that allows actin–myosin interaction, and as a result, contraction.

Mutations in the cTnI gene *TNNI3* account for ∼3 to 5% of genotyped families with HCM (5, 6). Human HCM causing cTnI mutation Gly203Ser is characterized by apical and septal hypertrophy, and in some cases supraventricular and ventricular arrhythmias (7, 8). In addition, HCM is characterized by myocyte remodeling, myofibril disarray, and altered energy metabolism (9). In mature cardiac muscle, cytoskeletal elements extend from the plasma membrane to Z disks and traverse cellular organelles, including t-tubules, sarcoplasmic reticulum, and mitochondria (10). In addition to modulating cell morphology, motility, intracytoplasmic transport, and mitosis (11, 12), cytoskeletal proteins also regulate the function of proteins in the plasma membrane. This includes the cardiac L-type calcium channel (I_Ca-L_), also known as the dihydropyridine channel. Using a murine model of the human Gly203Ser mutation (*cTnI-G203S*), we previously identified a role for the I_Ca-L_ in the development of HCM (13). We find that the cytoskeletal disarray is associated with a “communication breakdown” between the I_Ca-L_ and mitochondria, resulting in mitochondrial dysfunction.

The cardiac I_Ca-L_ is comprised of α_1C_, α_2_δ, and β_2_ subunits. The α_1C_ subunit forms the pore of the channel, which regulates ion conductance and voltage sensing (14). The β_2_ subunit is bound to the cytoplasmic I-II linker of the α_1C_ subunit (α-interaction domain, AID) (14, 15), and is anchored to F-actin via AHNAK (16). The β_2_ subunit regulates activation and inactivation kinetics of the channel (17). There is good evidence that I_Ca-L_ kinetics can also be influenced by alterations in F-actin organization (16, 18, 19). Furthermore, cytoskeletal proteins also interact directly with mitochondria by binding to outer mitochondrial docking proteins that can regulate mitochondrial function and energetics (11, 20, 21).

It is well known that calcium influx through I_Ca-L_ is critical to maintaining cardiac excitation and contraction. We have previously demonstrated that I_Ca-L_ also plays an important role in regulating mitochondrial function, and that this involves both calcium-dependent and calcium-independent mechanisms (22, 23). Activation of I_Ca-L_ with voltage-clamp of the plasma membrane or with the dihydropyridine receptor agonist BayK(−) is sufficient to increase intracellular and mitochondrial calcium, NADH production, superoxide production, and metabolic activity in *wt* cardiac myocytes, in a calcium-dependent manner (22). Activation of I_Ca-L_ also causes an increase in mitochondrial membrane potential (Ψ_m_), in a calcium-independent manner (22). This response is attenuated in the presence of F-actin depolymerizing agents, indicating that the response is in part dependent on an interaction between I_Ca-L_ and mitochondria, via F-actin (22). Immobilizing the I_Ca-L_ β_2_ subunit with a peptide derived specifically against the cardiac I_Ca-L_ AID (AID-TAT peptide) also attenuates the response (22). These findings indicate that I_Ca-L_ influences mitochondrial function through a structural–functional communication between I_Ca-L_ and mitochondria via the cytoskeletal network, following conformational changes in I_Ca-L_ that occur on a beat-to-beat basis.

Myocytes isolated from *cTnI-G203S* mice, which demonstrate characteristic features of HCM—including hypertrophy, hypercontractility, myofibril disarray, and interstitial fibrosis (7, 13)—exhibit altered communication between I_Ca-L_ and mitochondria, and altered metabolic activity. Specifically, *cTnI-G203S* myocytes exhibit a faster I_Ca-L_ inactivation rate, and increased Ψ_m_ and mitochondrial metabolic activity [consistent with the human condition (24)] in response to activation of I_Ca-L_ (13). It is important to note that these alterations also occur in myocytes isolated from hearts of *cTnI-G203S* mice that have not yet developed the cardiomyopathy, indicating that alterations in I_Ca-L_ kinetics and metabolic activity precede development of the cardiomyopathy.

We have previously demonstrated that AID-TAT peptide slows the inactivation rate of the cardiac I_Ca-L_, and attenuates elevated Ψ_m_ in response to activation of I_Ca-L_ in *wt* myocytes (22, 25, 26). We have also established that AID-TAT improves contractility and prevents the development of cardiac hypertrophy following coronary artery occlusion in rats (26). Importantly, this occurs without decreasing blood pressure or altering cardiac myocyte calcium influx (26). Therefore, the AID region of I_Ca-L_ is an attractive target for restoring mitochondrial function and preventing HCM. Here we hypothesized that targeting the cardiac I_Ca-L_ may be effective in preventing HCM in *cTnI-G203S* mice expressing the human cTnI mutation Gly203Ser by restoring mitochondrial function.

## Results

### Treatment of *cTnI-G203S* Cardiac Myocytes with AID-TAT Restores Mitochondrial Metabolic Activity.

**Fig. 1.**
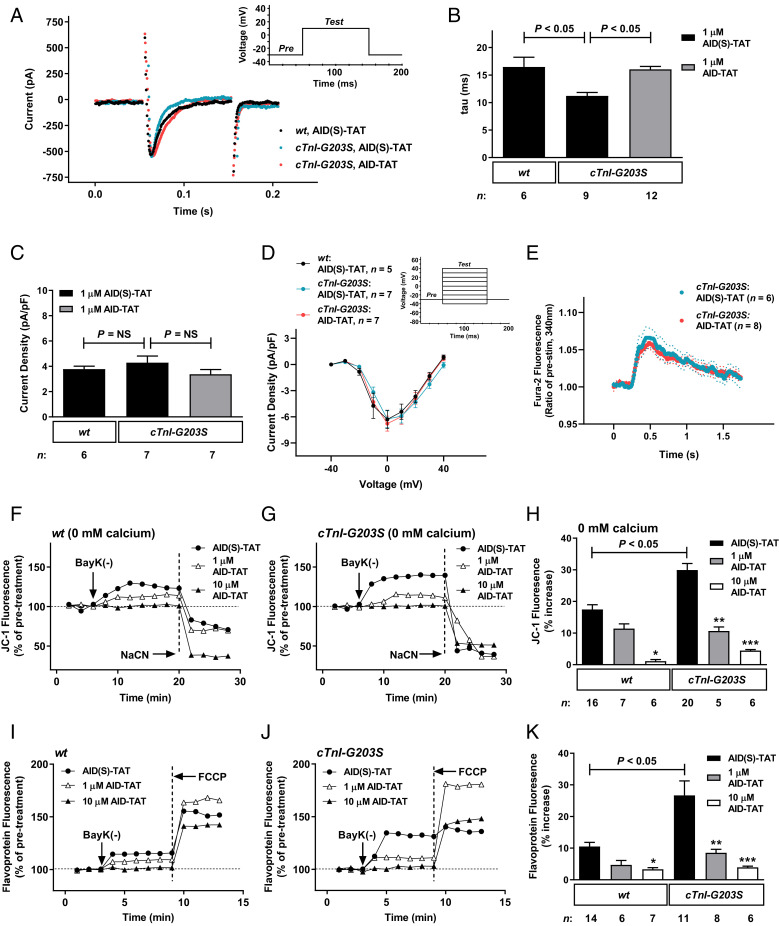
In vitro exposure of *cTnI-G203S* myocytes to AID-TAT peptide restores I_Ca-L_ kinetics, Ψ_m_ and flavoprotein oxidation in response to activation of I_Ca-L_. (*A*–*E*) I_Ca-L_ kinetics and intracellular calcium handling recorded in *wt* and *cTnI-G203S* cardiac myocytes in the presence of 1 μM AID(S)-TAT or AID-TAT. (*A*) Representative I_Ca-L_ current traces. (*Inset*) Pulse protocol. (*B* and *C*) Mean ± SEM of rate of inactivation (tau) (*B*) and current density (*C*) for all myocytes. (*D*) Current/voltage (*I-V*) relationships (*P* = NS, not significant). (*Inset*) Pulse protocol. (*E*) Calcium transients recorded in *cTnI-G203S* cardiac myocytes loaded with Fura-2, in the presence of 1 μM AID(S)-TAT or AID-TAT, presented as mean ± SEM (dashed line indicates SEM) (*P* = NS). Statistical significance determined by the Kruskal–Wallis test (*B* and *C*), two-way ANOVA with Geisser–Greenhouse correction (*D*), or the two-stage linear step-up method of Benjamini, et al. (68) (*E*). (*F*–*K*) Representative ratiometric JC-1 (*F*–*H*) and flavoprotein (*I*–*K*) fluorescence recorded from *wt* and *cTnI-G203S* myocytes before and after exposure to 10 μM BayK(−) in the presence of either 1 μM AID(S)-TAT, 1 μM AID-TAT, or 10 μM AID-TAT. JC-1 studies were performed under calcium-free conditions (0 mM calcium). Arrows indicate addition of drugs. To confirm signals were mitochondrial in origin, NaCN (40 mM) was applied at the end of each JC-1 experiment to collapse Ψ_m_. FCCP (50 μM) was applied at the end of each flavoprotein experiment to increase flavoprotein oxidation. Mean ± SEM of JC-1 (*H*) and flavoprotein (*K*) fluorescence for all myocytes (*n*) exposed to BayK in the presence of AID(S)-TAT or AID-TAT as indicated. *P < 0.05 compared with *wt* AID(S)-TAT, **P < 0.01 compared with *cTnI-G203S* AID(S)-TAT, ***P < 0.001 compared with *cTnI-G203S* AID(S)-TAT as determined by Kruskal–Wallis tests.

We characterized the effect of in vitro treatment of *cTnI-G203S* myocytes with AID-TAT on I_Ca-L_ kinetics and calcium handling using the whole-cell patch-clamp technique. Consistent with previous findings, inactivation rate of the current was significantly faster in *cTnI-G203S* myocytes exposed to inactive AID(S)-TAT peptide, compared to *wt* myocytes (Fig. 1 *A* and *B*). No difference in I_Ca-L_ current density was recorded in *cTnI-G203S* versus *wt* myocytes (Fig. 1 *C* and *D*), indicating that channel expression is not altered in *cTnI-G203S* myocytes. However, *cTnI-G203S* myocytes exposed to active AID-TAT restored I_Ca-L_ inactivation rate to *wt* levels (Fig. 1 *A* and *B*), with no significant alteration in current density or cellular calcium handling recorded (Fig. 1 *C*–*E*). These data indicate that the AID-TAT peptide regulates movement of the I_Ca-L_ β_2_ subunit, without affecting channel expression or calcium conductance.

We have previously demonstrated that *cTnI-G203S* myocytes exhibit increased Ψ_m_ and mitochondrial metabolic activity, consistent with the human condition (24), in response to activation of I_Ca-L_ (13). We have also shown that in vitro exposure of *wt* cardiac myocytes to 1 or 10 μM AID-TAT peptide prevents elevated Ψ_m_ and mitochondrial metabolic activity in response to activation of I_Ca-L_ (22, 25, 26). Here, we began by investigating the effect of in vitro exposure of myocytes isolated from hearts of 10-wk-old *cTnI-G203S* mice to 1 or 10 μM AID-TAT peptide on restoring Ψ_m_ and mitochondrial metabolic activity.

In order to examine the effect of AID-TAT peptide on the structural–functional communication between I_Ca-L_ and mitochondria, assessment of Ψ_m_ was performed under calcium-free conditions. Although ATP production is a calcium-dependent process, Ψ_m_ remains highly polarized (>170 mV) under conditions of low intracellular calcium (0 to 535 nM) (27, 28). Consistent with this, activation of I_Ca-L_ [by voltage-clamp, application of high K^+^ solution or BayK(−)] in guinea-pig ventricular myocytes incubated in calcium-free Hepes-buffered solution (≥3 h, supplemented with EGTA), followed by intracellular perfusion with EGTA and BAPTA, still yields an increase in Ψ_m_ (22). The response is dependent on an intact cytoskeletal architecture because depolymerization of actin with Latrunculin A attenuates the response (22). Similar findings have been observed in adult mouse cardiac myocytes (13, 25, 29).

We utilized I_Ca-L_ agonist BayK(−) to report mitochondrial function. Consistent with previous results, we find that *cTnI-G203S* cardiac myocytes exposed to calcium-free and EGTA containing HBS (calcium-free HBS) for at least 3 h exhibited a significantly larger increase in Ψ_m_ following application of BayK(−) in the presence of AID(S)-TAT peptide, assessed as changes in JC-1 fluorescence, compared to *wt* myocytes (Fig. 1 *F*–*H*) (13). In vitro exposure of myocytes to 1 μM AID-TAT peptide attenuated the response in *cTnI-G203S* but not *wt* myocytes, while 10 μM AID-TAT significantly attenuated the response in both *cTnI-G203S* and *wt* myocytes. Exposure of *cTnI-G203S* myocytes to 10 μM AID-TAT further attenuated elevated Ψ_m_ compared to 1 μM (*P* < 0.001 vs. *P* < 0.01) (Fig. 1 *G* and *H*).

We also examined changes in metabolic activity induced by activation of I_Ca-L_ in the myocytes. Metabolic activity is dependent upon oxygen consumption and electron flow down the inner mitochondrial membrane (30). Therefore, we examined alterations in mitochondrial electron transport in intact cardiac myocytes by measuring alterations in flavoprotein oxidation (as autofluorescence). Consistent with previous findings, we show that *cTnI-G203S* myocytes exhibited a significant increase in flavoprotein oxidation following exposure to BayK(−) in the presence of inactive AID(S)-TAT peptide, compared to *wt* myocytes (Fig. 1 *I*–*K*) (13). In vitro exposure of myocytes to 1 μM AID-TAT peptide attenuated the response in *cTnI-G203S* but not *wt* myocytes, while 10 μM AID-TAT significantly attenuated the response in both *cTnI-G203S* and *wt* myocytes. Exposure of *cTnI-G203S* myocytes to 10 μM AID-TAT further attenuated elevated mitochondrial metabolic activity compared to 1 μM (*P* < 0.001 vs. *P* < 0.05) (Fig. 1 *J* and *K*). Overall, we find that in vitro exposure of *cTnI-G203S* myocytes to 10 μM AID-TAT more effectively attenuates elevated Ψ_m_ and mitochondrial metabolic activity in response to activation of I_Ca-L_, compared to 1 μM AID-TAT (Fig. 1 *H* and *K*).

### AID-TAT Peptide Targets the Heart When Administered In Vivo.

**Fig. 2.**
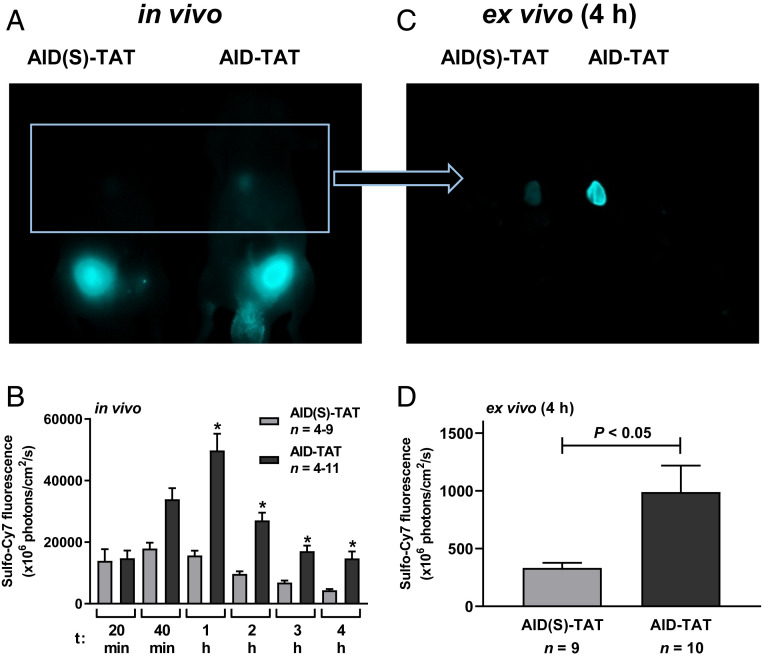
Cardiac uptake of AID-TAT peptide is significantly greater than AID(S)-TAT when administered in vivo. (*A*) Representative whole animal Cy7 fluorescent images from adult BALB/c nude mice administered a single 10-μM bolus dose of AID(S)-TAT-Cy7 or AID-TAT-Cy7, 4 h posttreatment. (*B*) Mean ± SEM of cardiac Cy7 fluorescence for all mice (*n*) administered AID(S)-TAT-Cy7 or AID-TAT-Cy7 acquired 20 and 40 min postinjection, and every hour after for up to 4 h. *P < 0.05 compared with AID(S)-TAT at matched time-points as determined by a Kruskal–Wallis test. (*C*) Representative ex vivo cardiac Cy7 fluorescent images from adult BALB/c nude mice administered AID(S)-TAT-Cy7 or AID-TAT-Cy7, 4 h posttreatment. (*D*) Mean ± SEM of cardiac Cy7 fluorescence for all ex vivo hearts (*n*), 4 h following administration of AID(S)-TAT-Cy7 or AID-TAT-Cy7. *P* < 0.05 as determined by a Mann–Whitney *U* test.

Prior to commencing an in vivo AID-TAT treatment regimen, we performed studies to examine cardiac uptake of the peptide. Eight-week-old BALB/c nude mice were administered a single 10-μM bolus dose of AID(S)-TAT-Cy7 or AID-TAT-Cy7. Maximal cardiac uptake of AID-TAT-Cy7 was achieved 1 h posttreatment, and remained significantly higher than AID(S)-TAT-Cy7 1 to 4 h postinjection (*P* < 0.05) (Fig. 2 *A* and *B*). At 4 h, hearts were extracted and ex vivo Cy7 fluorescence assessed. Consistent with in vivo findings, ex vivo AID-TAT-Cy7 fluorescence was significantly greater than AID(S)-TAT-Cy7 (Fig. 2 *C* and *D*).

**Fig. 3.**
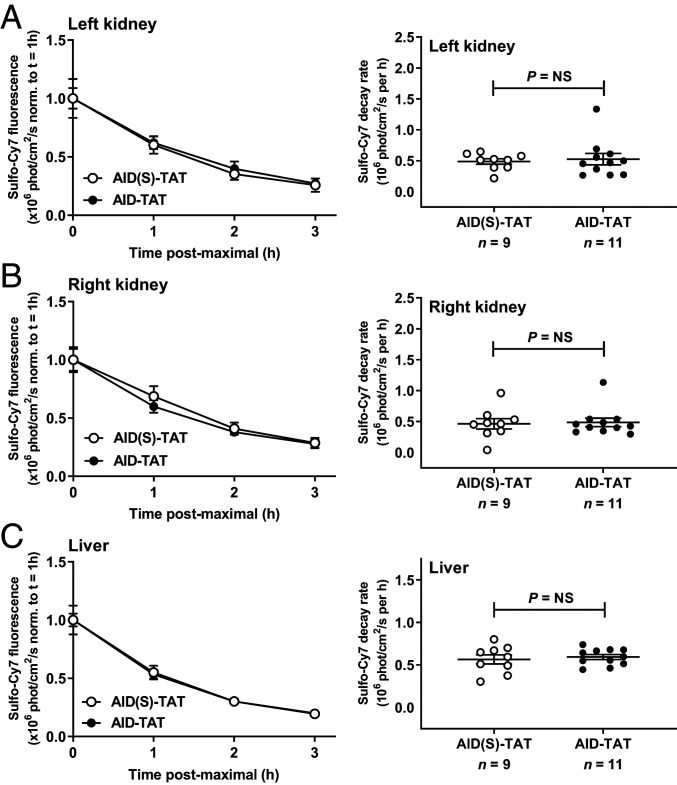
AID-TAT peptide is not retained by the kidneys or liver when administered in vivo. Clearance of AID-TAT and AID(S)-TAT via the left kidney (*A*), right kidney (*B*), and liver (*C*) for all mice administered AID(S)-TAT-Cy7 (*n* = 9) or AID-TAT-Cy7 (*n* = 11) over time (1 to 4 h postinjection). *Insets*, *Right*: Mean ± SEM of decay rates (exponential decrease) in Cy7 for all mice (*n*) over time (*t* = 1 to 4 h). Statistical significance determined by Mann–Whitney *U* tests (*A* and *B*) or unpaired *t* test with Welch’s correction (*C*). NS, not significant.

We also assessed bio-distribution, and the rate of clearance of the peptide from the kidneys and liver. Bio-distribution studies showed that AID-TAT-Cy7 uptake was significantly greater in the heart versus AID(S)-TAT-Cy7 (*t* = 1 h) (*SI Appendix*, Fig. S1*A*). No significant difference in uptake was recorded in the kidneys, liver, or bladder (*SI Appendix*, Fig. S1 *B*–*E*). Additionally, no significant difference in Cy7 decay was recorded in mice treated with AID-TAT versus AID(S)-TAT in the left or right kidney, or the liver (Fig. 3 *A*–*C*). These data indicate that AID-TAT is rapidly taken up by the heart versus AID(S)-TAT, and is not retained by the kidneys or liver when administered in vivo.

### In Vivo Treatment of Precardiomyopathic *cTnI-G203S* Mice with AID-TAT Restores Metabolic Activity.

Previous studies have identified that *cTnI-G203S* myocytes exhibit increased Ψ_m_ and mitochondrial metabolic activity, consistent with the human condition (24), in response to activation of I_Ca-L_ (13). These alterations precede development of the cardiomyopathy. Therefore, to develop a preventative therapy, we investigated the efficacy of treating precardiomyopathic *cTnI-G203S* mice with 10 μM AID-TAT on restoring cardiac Ψ_m_ and mitochondrial metabolic activity. Twenty-week-old precardiomyopathic *cTnI-G203S* mice (as evidenced by echocardiography) (*SI Appendix*, Table S1) were treated with 10 μM AID-TAT via intraperitoneal injection for 5 wk. It is assumed that AID-TAT lasts 3 to 4 d, consistent with the turnover rate of the I_Ca-L_ protein (31). Therefore, mice were administered AID-TAT peptide (10 μM) three times per week for 5 wk (3×/wk/5 wk).

**Fig. 4.**
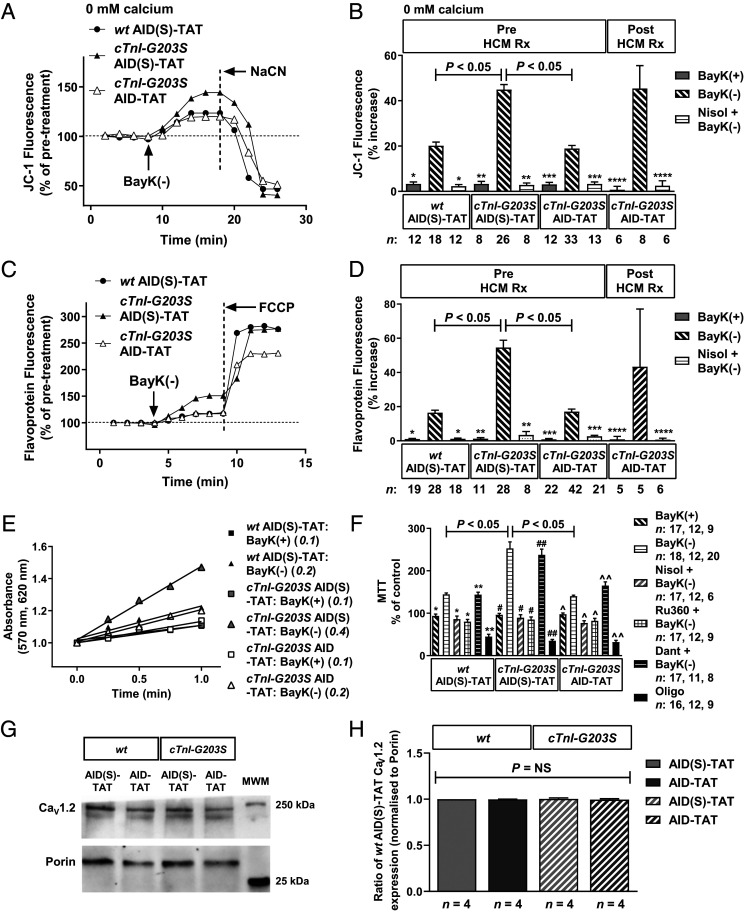
In vivo treatment of precardiomyopathic *cTnI-G203S* mice to AID-TAT peptide restores cellular Ψ_m_ and mitochondrial metabolic activity in response to activation of I_Ca-L_. Representative ratiometric JC-1 (*A*) and flavoprotein (*C*) fluorescence recorded from myocytes isolated from *wt* and *cTnI-G203S* mice treated with AID(S)-TAT or AID-TAT (10 μM, 3×/wk/5 wk), recorded before and after exposure to 10 μM BayK(−). JC-1 studies were performed under calcium-free conditions (0 mM calcium). Arrows indicate addition of drugs. NaCN: 40 mM, FCCP: 50 μM. Mean ± SEM of JC-1 (*B*) and flavoprotein (*D*) fluorescence for all myocytes (*n*) treated with AID(S)-TAT or AID-TAT, before or after the onset of HCM (Pre HCM Rx and Post HCM). Myocytes were exposed to BayK(+), BayK(−) or 15 μM nisoldipine (Nisol) as indicated. *P < 0.05 compared with *wt* AID(S)-TAT [BayK(− )], **P < 0.05 compared with *cTnI-G203S* AID(S)-TAT [BayK(−)], ***P < 0.05 compared with *cTnI-G203S* AID-TAT [BayK(− )], ****P < 0.05 compared with *cTnI-G203S* AID-TAT [BayK(−)] (Post-HCM Rx). (*E*) Formation of formazan measured as change in absorbance in myocytes from *wt* and *cTnI-G203S* mice recorded after addition of BayK(+) or BayK(−). Italicized values indicate slope. (*F*) Mean ± SEM of changes in absorbance for all myocytes (*n*) exposed to BayK(+), BayK(−), nisoldipine (Nisol), 15 µM Ru360, 20 µM dantrolene (Dant), or 20 µM oligomycin (Oligo) as indicated. *n* reported in succession for each experimental group [*wt* AID(S)-TAT, *cTnI-G203S* AID(S)-TAT and *cTnI-G203S* AID-TAT, respectively]. *P < 0.05 compared with *wt* AID(S)-TAT [BayK(−)], **P < 0.05 compared with *wt* AID(S)-TAT [BayK(+)], ^#^*P* < 0.05 compared with *cTnI-G203S* AID(S)-TAT [BayK(−)], ^##^*P* < 0.05 compared with *cTnI-G203S* AID(S)-TAT [BayK(+)], ^*P* < 0.05 compared with *cTnI-G203S* AID-TAT [BayK(−)], ^^*P* < 0.05 compared with *cTnI-G203S* AID-TAT [BayK(+)]. (*G* and *H*) Immunoblot analysis of I_Ca-L_ protein expression performed on total heart homogenate pooled from 4 *wt* or *cTnI-G203S* mice treated with AID(S)-TAT or AID-TAT (10 μM, 3×/wk/5 wk). (*G*) Representative immunoblots probed with I_Ca-L_ α_1C_ subunit antibody (Ca_V_1.2), then porin monoclonal antibody. (*H*) Densitometry analysis of Ca_V_1.2 protein expression presented as a ratio of *wt* AID(S)-TAT expression, normalized to associated porin expression. *n* = experimental repeats. All statistical significance was determined by Kruskal–Wallis tests. NS, not significant.

Myocytes isolated from precardiomyopathic *cTnI-G203S* mice treated with AID(S)-TAT exhibited a significantly larger increase in Ψ_m_ following exposure to BayK(−), compared to *wt* myocytes (Fig. 4 *A* and *B*). Treatment of precardiomyopathic *cTnI-G203S* mice with AID-TAT reduced this response to a level comparable to that observed in *wt* myocytes (Fig. 4 *A* and *B*). All BayK(−)-induced responses could be attenuated with application of I_Ca-L_ antagonist nisoldipine, confirming that I_Ca-L_ mediated the response (Fig. 4*B*). Application of the (+)enantiomer of BayK that does not act as an agonist [BayK(+)] did not significantly alter Ψ_m_ in myocytes isolated from *wt* or *cTnI-G203S* mice treated with AID(S)-TAT or AID-TAT (Fig. 4*B*).

Similarly, myocytes isolated from precardiomyopathic *cTnI-G203S* mice treated with AID(S)-TAT exhibited a significantly larger increase in flavoprotein oxidation following exposure to BayK(−), compared to *wt* myocytes (Fig. 4 *C* and *D*). Treatment of precardiomyopathic *cTnI-G203S* mice with AID-TAT reduced this response to a level comparable to that observed in *wt* myocytes (Fig. 4 *C* and *D*). All BayK(−)-induced responses could be attenuated with nisoldipine, confirming that I_Ca-L_ mediated the response (Fig. 4*D*). Application of BayK(+) did not significantly alter Ψ_m_ in myocytes isolated from *wt* or *cTnI-G203S* mice treated with AID(S)-TAT or AID-TAT (Fig. 4*D*).

Myocytes isolated from precardiomyopathic *cTnI-G203S* mice treated with AID(S)-TAT also exhibited a significantly larger increase in metabolic activity, assessed as formation of formazan from 3-(4,5-Dimethyl-2-thiazolyl)-2,5-diphenyl-2H-tetrazolium bromide (MTT), following exposure to BayK(−), compared to *wt* myocytes (Fig. 4 *E* and *F*). Treatment of precardiomyopathic *cTnI-G203S* mice with AID-TAT reduced this response to a level comparable to that observed in *wt* myocytes (Fig. 4 *E* and *F*). All BayK(−)-induced responses could be attenuated with application of nisoldipine or mitochondrial calcium uniporter blocker Ru360 (mitochondrial calcium uptake), but not with ryanodine receptor blocker dantrolene (sarcoplasmic reticulum calcium release) (Fig. 4*F*). Application of BayK(+) did not significantly alter metabolic activity in myocytes isolated from *wt* or *cTnI-G203S* mice treated with AID(S)-TAT or AID-TAT (Fig. 4 *E* and *F*). Oligomycin induced a significant decrease in metabolic activity in all myocytes demonstrating the myocytes were metabolically active (Fig. 4*F*) (30). We probed immunoblots of total heart homogenate pooled from precardiomyopathic *wt* or *cTnI-G203S* mice treated with AID(S)-TAT or AID-TAT (10 μM, 3×/wk/5 wk) with anti-Ca_V_1.2 antibody. Densitometry analysis confirmed no alteration in channel expression between any group (Fig. 4 *G* and *H*). Overall, these data indicate that in vivo treatment of precardiomyopathic *cTnI-G203S* mice with AID-TAT restores cardiac Ψ_m_ and mitochondrial metabolic activity to *wt* levels.

### In Vivo Treatment of *cTnI-G203S* Mice with Established Cardiomyopathy with AID-TAT Does Not Restore Metabolic Activity.

We investigated the efficacy of treating *cTnI-G203S* mice with established cardiomyopathy with AID-TAT on restoring cardiac Ψ_m_ and mitochondrial metabolic activity. Thirty-week-old cardiomyopathic *cTnI-G203S* mice (as evidenced by echocardiography) (*SI Appendix*, Table S1) were treated with AID-TAT (10 μM, 3×/wk/5 wk). Myocytes isolated from cardiomyopathic *cTnI-G203S* mice treated with AID-TAT exhibit a significant increase in Ψ_m_ and flavoprotein oxidation following exposure to BayK(−), to a level comparable to that observed in *cTnI-G203S* mice treated with AID(S)-TAT (Fig. 4 *B* and *D*). These data indicate that treatment of *cTnI-G203S* mice with established cardiomyopathy with this AID-TAT treatment regimen does not restore cardiac Ψ_m_ or mitochondrial metabolic activity to *wt* levels.

### Assessment of Myocardial Substrate Metabolism in *cTnI*-*G203S* Mice.

While the healthy adult heart utilizes long-chain fatty acid oxidization as a primary source of energy, hypertrophic and failing hearts shift toward glucose and lactate metabolism (32). The transition from oxidative fatty acid to glucose metabolism is thought to be associated with cardiac pathological remodeling (33). We performed targeted metabolomic assessment of whole heart tissue from *wt* and *cTnI-G203S* mice to obtain a global view of metabolic pathway perturbations associated with this mutation.

**Fig. 5.**
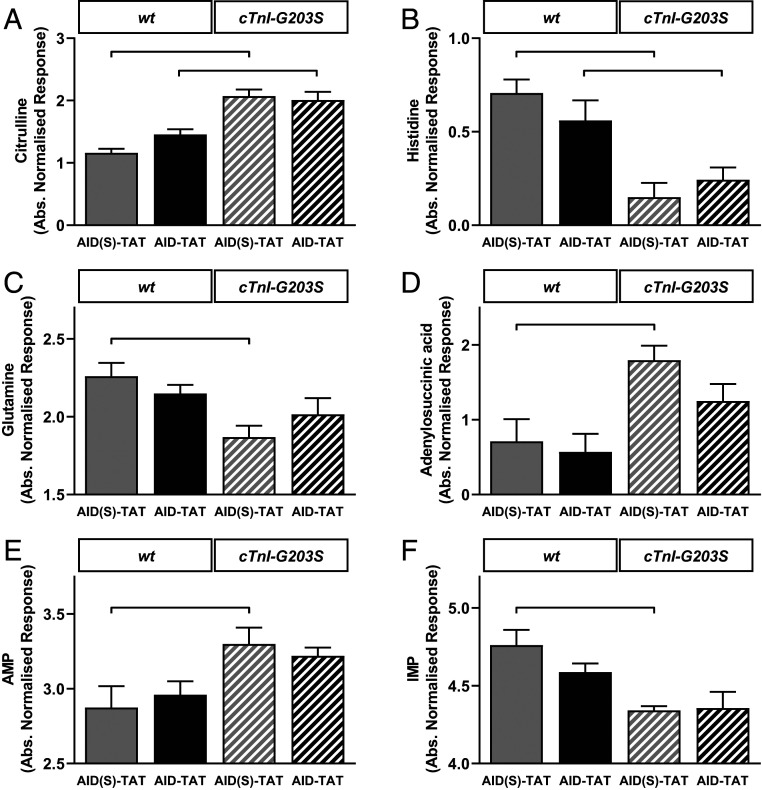
Metabolomics assessment of whole heart tissue reveals alterations in key metabolic pathways in the *cTnI-G203S* heart. Mean ± SEM of biochemical intermediates found to be significantly altered between *wt* and *cTnI-G203S* murine heart extracts by one-way ANOVA (false-discovery rate-adjusted *P* < 0.05). Between-group Fisher’s least significant difference post hoc significant differences are denoted with a bracketed line (adjusted *P* < 0.05) for citrulline (*A*), histidine (*B*), glutamine (*C*), adenylosuccinic acid (*D*), AMP (*E*), and IMP (*F*). *n* = 16 treated mice (four per treatment group).

Data quality was assessed numerically as well as visually using principal component analysis (*SI Appendix*, Fig. S2). Subsequent univariate analysis of the heart tissue metabolome revealed general differences between *wt* and *cTnI-G203S* mice. These differences centered around 26 significant biochemical intermediates (*P* < 0.05), with 14 of these deemed significant after correction for multiplicity (*SI Appendix*, Table S2). Analysis across the four experimental groups by one-way ANOVA resulted in 17 intermediates being significantly different across the groups (*SI Appendix*, Table S3). Three were deemed significant after correction for multiplicity. These included citrulline, histidine, and citric acid (false-discovery rate-corrected *P* < 0.05). The data were then mined (by correlation) for specific patterns of change related to the four groups. Significant changes were observed between AID(S)-TAT treated *cTnI-G203S* versus *wt* mice. Similar to ANOVA findings, these included citrulline (uric acid cycle, *P* < 0.00001), histidine (amino acid metabolism, *P* < 0.0002), glutamine (amino acid metabolism, *P* < 0.007), adenylosuccinic acid (purine metabolism, *P* = 0.003), adenosine 5-monophosphate (AMP, purine metabolism, amino acid metabolism, *P* < 0.004), and inosine 5-monophosphate (IMP, purine metabolism, amino acid metabolism, *P* = 0.001) (Fig. 5). Intermediates showing the largest differences between *wt* and *cTnI-G203S* hearts across all treatments were used as input for metabolite set enrichment analysis to delineate the metabolic pathways most represented as being modulated. These intermediates were involved mainly in amino acid and purine metabolism, as well as in the citric and uric acid cycles (*SI Appendix*, Fig. S3), with a number of metabolites being enriched in more than one pathway (*SI Appendix*, Table S4). Overall, alterations in amino acid and purine metabolism appear to occur in the *cTnI-G203S* heart.

### In Vivo Treatment of Precardiomyopathic *cTnI*-*G203S* Mice with AID-TAT Prevents HCM.

**Fig. 6.**
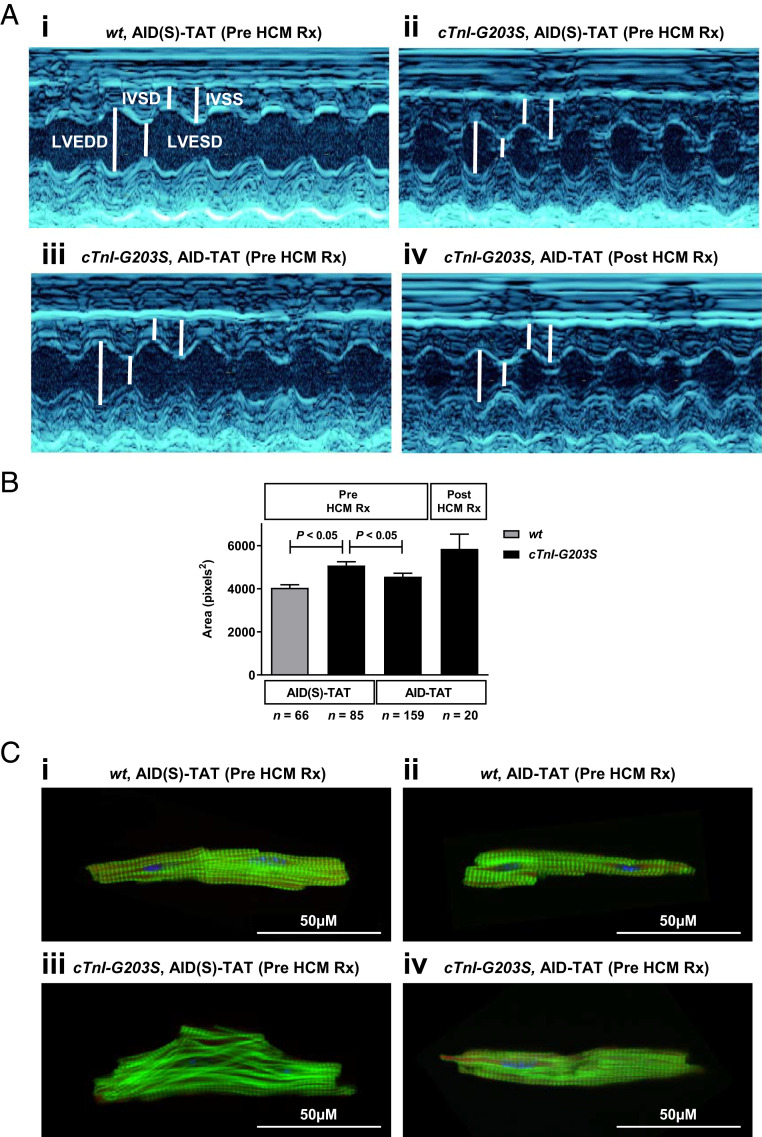
In vivo treatment of precardiomyopathic *cTnI-G203S* mice to AID-TAT peptide prevents development of cardiac hypertrophy. (*A*, *i–iv*) Representative images of echocardiographic measurements from *wt* and *cTnI-G203S* mice treated with AID(S)-TAT or AID-TAT (10 μM, 3×/wk/5 wk), before or after the onset of HCM (Pre HCM Rx and Post HCM Rx, respectively). (*B*) Mean ± SEM of cell size from myocytes isolated from *wt* and *cTnI-G203S* mice treated with AID(S)-TAT or AID-TAT (10 μM, 3×/wk/5 wk), before or after the onset of HCM (Pre HCM Rx and Post HCM Rx, respectively). *P* < 0.05 as determined by a Kruskal–Wallis test. (*C*) Representative confocal images of intact cardiac myocytes isolated from *wt* mice treated with 10 μM AID(S)-TAT (*i*, *n* = 10) or AID-TAT (*ii*, *n* = 8), and *cTnI-G203S* mice treated with 10 μM AID(S)-TAT (*iii*, *n* = 17) or AID-TAT (*iv*, *n* = 12), prior to the onset of HCM (Pre HCM Rx) as indicated. F-actin shown in green (phalloidin), nuclei shown in blue (DAPI).

We examined the efficacy of treating precardiomyopathic *cTnI-G203S* mice with AID-TAT on development of the cardiomyopathy. We performed serial echocardiography on *wt* and *cTnI-G203S* mice before and after treatment with AID(S)-TAT or AID-TAT (10 μM, 3×/wk/5 wk). Twenty-week-old *cTnI-G203S* mice exhibited no significant alterations in any echocardiographic parameter compared with age-matched *wt* mice, indicating the mice were precardiomyopathic (*SI Appendix*, Table S1). Consistent with the development of HCM, *cTnI-G203S* mice treated with AID(S)-TAT developed a significant decrease in left ventricular end diameter (diastolic diameter, LVEDD and systolic diameter, LVESD), and a significant increase in interventricular septum (diastole septum, IVSD and systole septum, IVSS), fractional shortening (FS), and heart weight to body weight (HW:BW), compared to *wt* mice treated with AID(S)-TAT (Fig. 6 *A*, *i* and *ii* and Table 1). In line with this, myocytes isolated from *cTnI-G203S* mice treated with AID(S)-TAT exhibited a significantly greater cell size compared to *wt* mice treated with AID(S)-TAT (Fig. 6*B*). However, *cTnI-G203S* mice treated with AID-TAT demonstrated a significant increase in LVEDD and LVESD, and a significant decrease in IVSD, IVSS, FS, and HW:BW compared to *cTnI-G203S* mice treated with AID(S)-TAT, to values comparable to those recorded in *wt* mice treated with AID(S)-TAT (Fig. 6 *A*, *iii* and Table 1). Consistent with this, myocytes isolated from *cTnI-G203S* mice treated with AID-TAT displayed a significantly lower cell size compared to *cTnI-G203S* mice treated with AID(S)-TAT (Fig. 6*B*). These data indicate that in vivo treatment of precardiomyopathic *cTnI-G203S* mice with AID-TAT prevents the development of HCM.

**Table 1.** Echocardiographic parameters of mice exposed to 10 μM AID(S)-TAT or AID-TAT

LVEDD (mm)	LVESD (mm)	FS (%)	LVDPW (mm)	LVSPW (mm)	IVSD (mm)	IVSS (mm)	HW:BW (mg/g)	HR (bpm)
Pre-HCM treatment (from 20 wk)								
25-wk-old *wt*, AID(S)-TAT (*n* = 4)								
3.56	2.31	35.08	1.26	1.42	0.76	0.86	4.33	450
±0.12	±0.06	±1.11	±0.08	±0.05	±0.01	±0.01	±0.07	±37
25-wk-old *cTnI-G203S*, AID(S)-TAT (*n* = 7)								
3.08*	1.62*	47.39*	1.31	1.43	0.85*	0.96*	5.14*	450
±0.06	±0.05	±0.81	±0.03	±0.04	±0.01	±0.02	±0.10	±22
25-wk-old *cTnI-G203S*, AID-TAT (*n* = 6)								
3.33^†^	2.06^†^	38.26^†^	1.23	1.37	0.73^†^	0.82^†^	4.70^†^	452
±0.06	±0.08	±1.61	±0.03	±0.03	±0.00	±0.01	±0.17	±26
Post-HCM treatment (from 30 wk)								
35-wk-old *cTnI-G203S*, AID-TAT (*n* = 5)								
3.18	1.67	48.48	1.26	1.43	0.95	1.05	5.81	450
±0.07	±0.05	±1.07	±0.03	±0.06	±0.03	±0.02	±0.21	±19

Values reported as mean ± SEM; bpm, beats per minute.

* *P* < 0.05 compared to 25-wk-old *wt* AID(S)-TAT.

^†^ *P* < 0.05 compared with 25-wk-old *cTnI-G203S* AID(S)-TAT as determined by Kruskal–Wallis tests.

We also examined the efficacy of AID-TAT treatment of *cTnI-G203S* mice with established HCM. Consistent with the development of HCM, 30-wk-old *cTnI-G203S* mice exhibited a significant decrease in LVEDD and LVESD, and a significant increase in IVSD, IVSS, left ventricular posterior wall (in diastole, LVDPW and in systole, LVSPW) and FS compared to 20-wk-old *cTnI-G203S* mice (*SI Appendix*, Table S1). Treatment of 30-wk-old *cTnI-G203S* mice with AID-TAT (10 μM, 3×/wk/5 wk) did not significantly improve echocardiographic parameters, or alter cell size, compared to *cTnI-G203S* mice treated with AID(S)-TAT (Fig. 6 *A*, *iv* and *B* and Table 1). These data indicate that AID-TAT treatment of *cTnI-G203S* mice with established HCM is not effective at reversing the cardiomyopathy.

### In Vivo Treatment of Precardiomyopathic *cTnI-G203S* Mice with AID-TAT Restores Cytoskeletal Organization.

There is good evidence that *cTnI-G203S* mice exhibit myofibril and mitochondrial disorganization (7, 13). We have previously shown that *cTnI-G203S* cardiac myocytes exhibit altered structural-functional communication between I_Ca-L_ and mitochondria via the cytoskeletal network (13). This is associated with the development of a hypermetabolic mitochondrial state that leads to the development of HCM. Here we assessed the effect of treatment of *cTnI-G203S* mice with AID-TAT on cytoskeletal organization. Consistent with a healthy myocardium, cardiac myocytes isolated from *wt* mice treated with AID(S)-TAT or AID-TAT (10 μM, 3×/wk/5 wk) demonstrate organized F-actin architecture (Fig. 6 *C*, *i* and *ii*). Myocytes isolated from *cTnI-G203S* mice treated with AID(S)-TAT exhibit F-actin disorganization (Fig. 6 *C*, *iii*). However, myocytes from *cTnI-G203S* mice treated with AID-TAT demonstrate F-actin organization comparable to *wt* myocytes (Fig. 6 *C*, *iv*). These findings indicate that in vivo treatment of precardiomyopathic *cTnI-G203S* mice with AID-TAT may restore cellular cytoskeletal organization.

### In Vivo Treatment of *cTnI-G203S* Mice with AID-TAT Is Not Toxic.

**Fig. 7.**
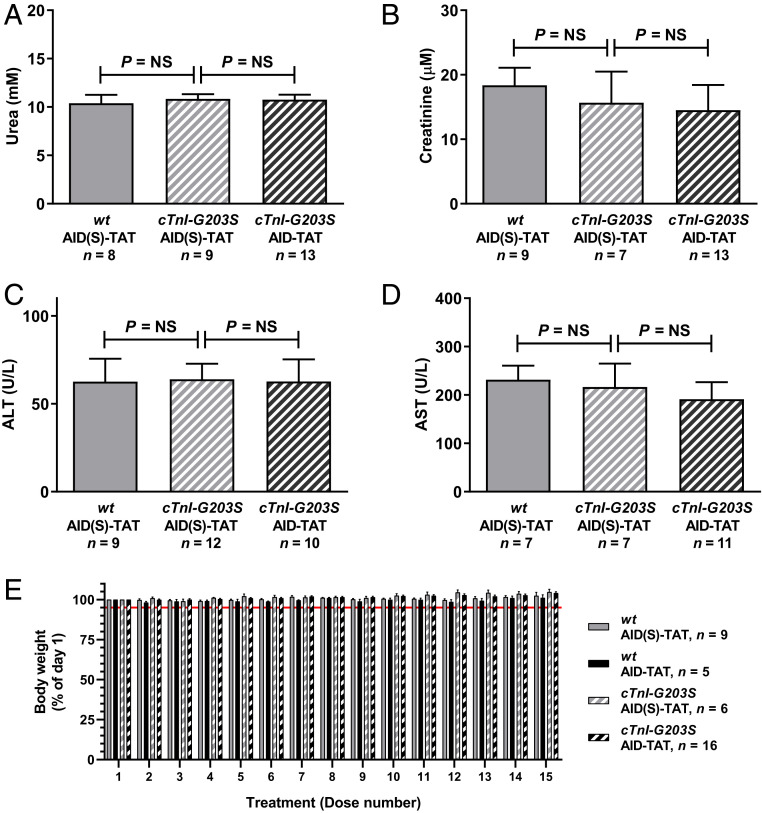
In vivo treatment of precardiomyopathic *cTnI-G203S* mice to AID-TAT peptide is not toxic. Mean ± SEM of urea (*A*), creatinine (*B*), ALT (*C*), and AST (*D*) concentrations from terminal serum from *wt* and *cTnI-G203S* mice treated with AID(S)-TAT or AID-TAT (10 μM, 3×/wk/5 wk) as indicated. *n* = number of mice. *P* = NS (not significant) determined by the Brown-Forsythe and Welch ANOVA test (*A*) or Kruskal–Wallis tests (*B*–*D*). (*E*) Mean ± SEM of body weight recorded from *wt* and *cTnI-G203S* mice treated with AID(S)-TAT or AID-TAT (10 μM, 3×/wk/5 wk, equaling 15 doses: 1 to 15) reported as a percentage (%) of day 1 BW. *n* = number of mice. Red line indicates 5% weight-loss threshold.

Upon completion of in vivo AID-TAT treatment regimen, terminal serum was collected and assessed for kidney and liver toxicity. No significant alterations in urea and creatinine, or alanine transaminase (ALT) and aspartate transaminase (AST) were measured (Fig. 7 *A*–*D*). Additionally, no significant reduction in BW was observed over the course of the treatment protocol (Fig. 7*E*). These data indicate that this treatment protocol is not toxic.

## Discussion

HCM occurs due to mutations in sarcomeric proteins. It is characterized by myofibril disorganization, altered energy metabolism, and myocyte remodeling (7, 9). Using a murine model of the human HCM causing cTnI mutation Gly203Ser (*cTnI-G203S*), we previously identified that this mutation is associated with a faster I_Ca-L_ inactivation rate, impaired functional communication between the channel and mitochondria, and increased mitochondrial metabolic activity (13) that is consistent with the human condition (24). Importantly, we identified that alterations in I_Ca-L_ kinetics and mitochondrial metabolic activity precede development of the cardiomyopathy. That is, impaired structural–functional communication between the I_Ca-L_ and mitochondria contributes to the development of HCM.

To date, clinical management of HCM has focused on treatment of symptoms. This includes the use of β-adrenergic receptor blockers and I_Ca-L_ antagonists that nonspecifically reduce contractile strength (3, 34). Despite varying degrees of efficacy relieving symptoms in patients, these approaches are often associated with side-effects and negative inotropic action (35). There are no currently available treatments that can prevent or reverse the cardiomyopathy. We have previously demonstrated a peptide that targets the cardiac I_Ca-L_ (AID-TAT) that improves contractility and prevents the development of cardiac hypertrophy following coronary artery occlusion in rats (26). Importantly, AID-TAT treatment does not alter blood pressure or cardiac myocyte calcium influx (26). This is because, unlike traditional I_Ca-L_ antagonists, AID-TAT targets the AID of the channel (immobilizing the β_2_ subunit), rather than the pore-forming α_1C_ subunit. The AID-TAT peptide slows the inactivation rate of I_Ca-L_ and decreases mitochondrial metabolic activity in a structural–functional manner (22, 25). We now demonstrate that application of AID-TAT to cardiac myocytes isolated from *cTnI-G203S* mice restores I_Ca-L_ inactivation rate to *wt* levels, without impacting on channel expression, calcium influx, or the calcium transient (Figs. 1 *A*–*E* and 4 *G*–*H*). Therefore, the AID region of I_Ca-L_ represents a viable target for restoring mitochondrial function and preventing the development of *cTnI-G203S* cardiomyopathy, without causing negative inotropic effects.

In this study, we investigated the efficacy of in vivo treatment of precardiomyopathic *cTnI-G203S* mice on restoring mitochondrial metabolic activity, and preventing HCM. Initial studies indicated that in vitro exposure of cardiac myocytes isolated from precardiomyopathic *cTnI-G203S* mice to 10 μM AID-TAT peptide more effectively attenuated elevated Ψ_m_ and mitochondrial metabolic activity in response to activation of I_Ca-L_ compared to 1 μM AID-TAT (Fig. 1). Given that the turnover rate of the I_Ca-L_ protein is ∼3 to 4 d (31), we investigated the efficacy of treating precardiomyopathic *cTnI-G203S* mice with 10 μM AID-TAT three times per week on restoring mitochondrial metabolic activity, and subsequently, preventing HCM. Initial studies indicated that in vivo, AID-TAT was specifically and efficiently taken up by the heart, and was not retained by the kidneys or liver (Figs. 2 and 3). Subsequent toxicity studies confirmed that this treatment regimen was not toxic (Fig. 7).

Patients with HCM often present with early hypercontractility that stems from a high-degree of actin–myosin cross-linking (36). Indeed, altered actin–myosin kinetics, specifically enhanced filament sliding, has been demonstrated to occur in models of *cTnI-G203S* (37, 38). Recent studies have investigated the efficacy of a cardiac-specific small-molecule, Mavacamten (MYK-461), that inhibits β-MHC/actin binding and subsequently reduces sarcomere force output and contractility (39, 40), as a potential HCM therapeutic. Treatment of precardiomyopathic mice expressing β-MHC mutations with MYK-461 has been shown to reduce left ventricular wall thickness, FS, and fibrosis, and improve myocyte organization compared to untreated mutant counterparts (39). The majority of MYK-461 efficacy has been in relieving obstructive HCM (41, 42) and nonobstructive HCM hypercontractility (43, 44). This is consistent with the mode of action of MYK-461. Here we find that administering precardiomyopathic *cTnI-G203S* mice AID-TAT completely restored Ψ_m_ and metabolic activity (Fig. 4) and prevented development of characteristic hypertrophy and hypercontractility (Fig. 6 *A* and *B* and Table 1). Based on the mode of action of AID-TAT peptide on I_Ca-L_ kinetics (Fig. 1 *A*–*E*) (22), we propose that application of this peptide restores the structural–functional communication between the I_Ca-L_ and mitochondria, thereby normalizing mitochondrial metabolic activity and preventing development of HCM.

There is good evidence that HCM patients exhibit elevated metabolic activity and increased myocardial stiffness (6, 24, 45–47). We propose that the decrease in myocardial stiffness observed in AID-TAT treated *cTnI-G203S* mice, evidenced by reduced FS (Table 1), may be associated with restored cytoskeletal organization (Fig. 6*C*); however, further studies directly quantifying cytoskeletal organization would be required to determine this conclusively. Interestingly, administering AID-TAT to *cTnI-G203S* mice with established cardiomyopathy did not effectively restore Ψ_m_ or metabolic activity (Fig. 4) or reverse the cardiomyopathy (Fig. 6 *A* and *B* and Table 1). These data suggest that an early-intervention approach may be efficacious in restoring altered metabolic activity and preventing subsequent *TnI-G203S* cardiomyopathy.

While the healthy adult heart utilizes long-chain fatty acid oxidization as a primary source of energy, hypertrophic and failing hearts shift toward glucose and lactate metabolism (32). To date, limited metabolomic studies have been performed in HCM hearts. However, one study on HCM patients carrying a cardiac myosin binding protein-C (*MYBPC3*) mutation (Q1061X) with HCM exhibited increased plasma triglycerides and branched-chain amino acids compared to control subjects without HCM (48). In line with this, we find that hearts from *cTnI-G203S* mice exhibit alterations in key metabolic pathways, such as increased glycolysis (2-phosphoglyceric acid and dihydroxyacetone phosphate) and tricarboxylic acid (TCA) cycle (citrate/isocitrate, succinate, aconitate) metabolism, as well as amino acid metabolism (especially glutamine) versus *wt* (Fig. 5 and *SI Appendix*, Tables S2–S4). Purine metabolism was also affected, with a decrease in IMP and aspartate, and concomitant increases in adenylosuccinic acid and AMP recorded (Fig. 5 and *SI Appendix*, Tables S2–S4), pointing to an increased requirement for adenine nucleotides for ATP production in *cTnI-G203S* hearts. These data are consistent with previous findings demonstrating an increase in adenylosuccinic acid and AMP in surgically or thiazolidinedione-induced cardiac hypertrophy (49, 50). Increases in adenylosuccinic acid and AMP production may also provide fumarate for anaplerotic refilling of the TCA cycle. Similarly, decreases in amino acids (histidine and glutamine) and perturbations in urea cycle intermediates (citrulline and argininosuccinic acid) were observed (Fig. 5 and *SI Appendix*, Tables S2–S4), as a possible consequence of transamination/deamination reactions. Deamination of histidine to urocanate (and subsequently glutamate), and glutamine to glutamate, may indicate a role for these amino acids in anaplerotic refilling of the TCA cycle (via glutamate to α-ketoglutarate) in *cTnI-G203S* hearts. These alternative intermediary pathways may play a compensatory role in *cTnI-G203S* hearts in order to meet the increased energy demands of the cell, as indicated by a hypermetabolic mitochondrial state (Figs. 1 *F*–*K* and 4 *A*–*F*) (13). Indeed, increased anaplerosis is thought to play a cardioprotective role in the development of pressure overload hypertrophy and heart failure (51–53). Given the mode of action of the AID-TAT peptide, we would not expect AID-TAT treatment to have direct effects on compensatory alterations in metabolic substrates observed in the *cTnI-G203S* mice (Fig. 5); however, long-term treatment may reestablish long-chain fatty acid oxidization as a primary source of energy production.

Overall, we postulate that metabolomic profiling may assist in identifying nonhypertrophic Gly203Ser mutation carriers who are at risk for developing HCM. Metabolites, such as those presented in Fig. 5, may represent useful biomarkers of the disease state. Additionally, we identify a nontoxic, preventative approach for the treatment of HCM resulting from cTnI mutation Gly203Ser. We speculate that utilizing AID-TAT may represent a viable means to restore structural–functional communication between I_Ca-L_ and mitochondria, normalize metabolic activity, and prevent the development of HCM. The AID region of the cardiac I_Ca-L_ is highly conserved (100%) between species, including rodents and humans (54, 55). Therefore, this approach may prove beneficial in the prevention of HCM in patients with identified Gly203Ser gene mutations.

While some similarities exist, different models of HCM appear to exhibit mutation-specific alterations in calcium handling, myofilament calcium sensitivity, and mitochondrial metabolic function (6). For example, we have previously demonstrated that *cTnI-G203S* mice, and *αMHC*^*403/+*^ mice expressing human HCM causing *MYH7* mutation Arg403Gln, exhibit similar alterations in I_Ca-L_ and mitochondrial metabolic activity (6, 29). However, these models of HCM display differences in calcium handling and myofilament calcium sensitivity. Mutation-specific mechanisms may contribute to the phenotypic variability observed in human HCM, and as a result, responsiveness to therapy. Indeed, clinical studies indicate that *MYBPC3* mutation carriers may be more responsive to I_Ca-L_ agonist diltiazem than *MYH7* mutation carriers (56). With this, further studies would be required to investigate the efficacy of AID-TAT on normalizing metabolic activity and preventing HCM in models of the disease resulting from mutations other than Gly203Ser to assess feasibility of translation to the clinical setting.

## Materials and Methods

### AID-TAT Peptide.

A peptide corresponding to the α_1C_–β_2_ interaction domain within the cytoplasmic I–II linker of the cardiac α_1C_ subunit (AID) was synthesized using the amino acid sequence, QQLEEDLKGYLDWITQAE (manufactured by Philip Thompson, Monash University, Melbourne, VIC, Australia) (57). A scrambled (inactive) control peptide [AID(S)] was also synthesized (QKILGEWDLAQYTDQELE). A cell-penetrating TAT sequence was tethered to the peptides via 6-aminohexanoic acid (RKKRRQRRR), to yield AID-TAT and AID(S)-TAT peptides.

### Animal Model.

Male mice expressing the human cTnI gene encoding the human disease-causing mutation *cTnI-G203S* were used for all studies. The mice develop hallmark features of HCM by 21 wk (7, 13). Male mice expressing the normal human cTnI gene were used as controls (*wt*). Ten-week-old mice were used for in vitro studies. For in vivo studies, 20- or 30-wk-old *cTnI-G203S* mice were treated with 10 μM AID-TAT or AID[S]-TAT via intraperitoneal injection, 3×/wk/5 wk. In vivo dose of AID-TAT or AID[S]-TAT (10 μM) was calculated based on blood volume, equating to ∼2 mg/kg total BW. Male animal models were used to eliminate potential differences in responses due to sex. Experiments were performed in a total of 21 *wt* (AID[S]-TAT), 6 *wt* (AID-TAT), 15 *cTnI-G203S* (AID[S]-TAT), and 26 *cTnI-G203S* (AID-TAT) mice. All animals were randomly assigned to treatment groups. All animal studies were approved by the Animal Ethics Committee of The University of Western Australia in accordance with the 2013 *Australian Code for the Care and Use of Animals for Scientific Purposes* (58).

### Isolation of Ventricular Myocytes.

Myocytes were isolated from *wt* and *cTnI-G203S* mice. Animals were anesthetized with pentobarbitone sodium (240 mg/kg) via intraperitoneal injection prior to excision of the heart. Cells were isolated as previously described (25, 59, 60). All in vitro studies were performed in freshly isolated myocytes at 37 °C. Detailed methods are provided in *SI Appendix*.

### Patch-Clamp and Calcium Transient Studies.

The whole-cell configuration of the patch-clamp technique was used to measure changes in I_Ca-L_ currents (13), and stimulate calcium transients in intact ventricular myocytes. Detailed methods are provided in *SI Appendix*.

### Measurement of In Vitro Mitochondrial Membrane Potential (Ψ_m_) and Mitochondrial Flavoprotein Oxidation.

Fluorescence was measured on a Hamamatsu Orca ER digital camera attached to an inverted Nikon TE2000-U microscope. Fluorescent indicator 5,5′,6,6′-tetrachloro-1,1′,3,3′-tetraethylbenzimidazolylcarbocyanine iodide was used to measure Ψ_m_ in cardiac myocytes, as previously described (JC-1, Molecular Probes) (61). Autofluorescence was used to measure flavoprotein oxidation in cardiac myocytes based on previously described methods (13, 29, 62). Responses to drugs were reported as a percentage increase from the basal average. Detailed methods are provided in *SI Appendix*.

### Assessment of In Vivo Cardiac Uptake and Bio-Distribution of AID-TAT Peptide.

Eight-week-old male BALB/c nude mice were used to assess in vivo cardiac uptake and bio-distribution of sulfo-Cyanine7-labeled AID(S)-TAT and AID-TAT (Cy7, W&J PharmaChem) using a CRi Maestro 2 multispectral imaging system (Cambridge Research and Instrumentation) based on previously described methods (63). All studies were performed in mice anesthetized with isoflurane (2 to 4%), followed by intraperitoneal injection of pentobarbitone sodium (240 mg/kg), as approved by the Animal Ethics Committee of The University of Western Australia in accordance with the 2013 *Australian Code for the Care and Use of Animals for Scientific Purposes* (58). Detailed methods are provided in *SI Appendix*.

### MTT Assay.

The rate of reduction of MTT (Sigma-Aldrich) to formazan by the mitochondrial electron transport chain was measured in intact mouse cardiac myocytes, as previously described (22, 25). Each *n* represents number of replicates for each treatment group from cardiac myocytes isolated from a total of six *wt* [AID(S)-TAT], four *cTnI-G203S* [AID(S)-TAT], and three (AID-TAT) mice. Detailed methods are provided in *SI Appendix*.

### Immunoblot of I_Ca-L_ Protein.

Immunoblot analysis of I_Ca-L_ protein expression was performed on total heart homogenate pooled from groups of four *wt* or *cTnI-G203S* mice treated with AID(S)-TAT or AID-TAT (10 μM, 3×/wk/5 wk). Blots were probed with rabbit polyclonal anti-Ca_V_1.2 (Alomone Labs, ACC-003; 1:200) and rabbit monoclonal anti-porin (Cell Signaling, #4661; 1:1,000) primary antibodies, then with goat anti-mouse IgG H&L (HRP) preadsorbed secondary antibody (Abcam, AB97040; 1:10,000). Detailed methods are provided in *SI Appendix*.

### Metabolomic Analysis of Whole Heart Tissue.

Whole hearts were processed and analyzed for perturbations in central metabolic pathways using targeted liquid chromatography-tandem mass spectrometry-based metabolomics. Detailed methods are provided in *SI Appendix*.

### Echocardiography.

Echocardiographic measurement of left ventricular function were performed on mice under light methoxyflurane anesthesia using an i13L probe on a Vivid 7 Dimension ultrasound system (GE Healthcare), as previously described (13, 29, 30). Each *n* represents the average of quantitative measurements from *wt* or *cTnI-G203S* mice for each treatment group. Detailed methods are provided in *SI Appendix*.

### Cell Size.

Cell size was determined as previously described (64). Detailed methods are provided in *SI Appendix*.

### Sample Preparation for Confocal Imaging.

Following completion of treatment regimens, myocytes were isolated and prepared for confocal imaging based on previously described methods (29). Detailed methods are provided in *SI Appendix*.

### Toxicity Parameters.

Mice were treated with 10 μM AID(S)-TAT or AID-TAT 3×/wk/5 wk (equaling 15 doses). BW was recorded prior to administration of each peptide dose, and reported as a percentage of day 1 BW. A 5 to 10% reduction in BW directed increased monitoring as recommended by The Animal Ethics Committee of The University of Western Australia in accordance with the 2008 *Guidelines to Promote the Wellbeing of Animals Used for Scientific Purposes* (65). Following completion of treatment regimen, mice were anesthetized and terminal blood collected. Serum was extracted and used to measure kidney and liver toxicity as previously described (66). Detailed methods are provided in *SI Appendix*.

### Statistical Analysis.

Results are reported as mean ± SEM. For nonparametric data, statistical significance was accepted at *P* < 0.05 using the Mann–Whitney *U* test or Kruskal–Wallis test for multiple comparisons. For parametric data, statistical significance was accepted at *P* < 0.05 using an unpaired *t* test with Welch’s correction, the Brown-Forsythe and Welch ANOVA test, or two-way ANOVA with Geisser–Greenhouse correction (GraphPad Prism v5.04). Metabolomics data were analyzed with SIMCA v15 (Sartorius Stedim Data Analytics) and MetaboAnalyst v4.0 (see *SI Appendix*, *Supporting Materials and Methods* for details) (67). Number of replicates and statistical comparisons are specified in figures and figure legends.

## Supplementary Material

Supplementary File

## Data Availability

All study data are included in the main text and *SI Appendix*.
