# Supplementary material for: Characterization and validation of a preventative therapy for hypertrophic cardiomyopathy in a murine model of the disease
Source: Proc Natl Acad Sci U S A. 2020 Aug 28;117(37):23113–24. doi: 10.1073/pnas.2002976117 (PMC7502707; doi:10.1073/pnas.2002976117)
Supplement: Supplementary File [file pnas.2002976117.sapp.pdf]

PNAS
www.pnas.org

Supplementary Information for

Characterization and validation of a preventative therapy for hypertrophic cardiomyopathy in a murine model of the disease.

Helena M. Viola[1]*, Ashay A. Shah[1], Victoria P.A. Johnstone[1], Henrietta Cserne Szappanos[1], Mark P. Hodson[2,3], Livia C. Hool[1,2]*

[1]School of Human Sciences (Physiology), The University of Western Australia, Crawley, WA, Australia. [2]Victor Chang Cardiac Research Institute, Sydney, NSW, Australia. [3]School of Pharmacy, University of Queensland, Woolloongabba, QLD, Australia.

*Corresponding author: Helena M. Viola and Livia C. Hool

Email: helena.viola@uwa.edu.au, livia.hool@uwa.edu.au

This pdf file includes:

Supplementary text
Figures S1 to S3
Tables S1 to S4
SI References

www.pnas.org/cgi/doi/10.1073/pnas.2002976117

Supporting Materials and Methods

Isolation of ventricular myocytes

Myocytes were isolated from *wt* and *cTnI-G203S* mice. Animals were anesthetized with pentobarbitone sodium (240 mg/kg) via intraperitoneal injection prior to excision of the heart. Cells were isolated as previously described (1-3). Mouse hearts were excised, cannulated onto a Langendorff apparatus via the aorta, and perfused with Krebs-Henseleit Buffer (KHB) containing (in mM): 120 NaCl, 25 $NaHCO_3$, 4.8 KCl, 2.2 $MgSO_4$, 1.2 NaH_2PO_4 and 11 glucose (pH = 7.35 with O_2/CO_2 at 37°C) for 4 min at 37°C. Hearts were then perfused with KHB supplemented with 2.4 mg/ml collagenase B for 3 min, followed by a further 8 min perfusion in the presence of 40 μM calcium. Following perfusion, aorta and atria were removed then ventricles teased apart and triturated to dissociate myocytes into suspension. Myocyte suspension was spun at 500 RPM for 3 min, supernatant discarded, and myocytes resuspended in calcium free Hepes-Buffered Solution (HBS) containing (in mM): 5.3 KCl, 0.4 $MgSO_4.7H_2O$, 139 NaCl, 5.6 $Na_2HPO_4.2H_2O$, 5 glucose, 20 Hepes and 2 glutamine (pH = 7.4 at 37°C) in the presence or absence of 3 mM EGTA (for 0 mM calcium experiments). For calcium containing experiments, calcium was titrated back to achieve a final extracellular concentration of 1.8 mM.

Patch-clamp and calcium transient studies

The whole-cell configuration of the patch-clamp technique was used to measure changes in I_{Ca-L} currents (4), and stimulate calcium transients in intact ventricular myocytes. Microelectrodes with tip diameters of 3-5 μm and resistances of 0.5-1.5 MΩ contained (in mM): CsCl 115, HEPES 10, EGTA 10, tetraethylammonium chloride 20, MgATP 5, Tris-GTP 0.1, phosphocreatine 10 and $CaCl_2$ 1 (pH adjusted to 7.2 at 37°C with CsOH). Currents were measured in extracellular modified Tyrode's solution containing (in mM): NaCl 140, CsCl 5.4, $CaCl_2$ 2.5, $MgCl_2$ 0.5, HEPES 5.5 and glucose 11 (pH adjusted to 7.6 with NaOH). Macroscopic currents were recorded using an Axopatch 200B voltage-clamp amplifier (Molecular Devices) with a Digidata 1440A interface and pClamp10.5 software (Molecular Devices). A Ag/AgCl electrode was used to ground the bath. Once the whole-cell configuration was achieved, the holding potential was set at –80 mV. Na^+ channels and T-type Ca^{2+} channels were inactivated by applying a 50 ms prepulse to –30 mV immediately before each test pulse. The time course of changes in Ca^{2+} conductance were monitored by applying a 100 ms test pulse to 10 mV once every 10 seconds. Kinetics of calcium current inactivation was analysed by fitting current decay after channel activation with an exponential function (yielding tau).

For calcium transient studies, microelectrodes contained (in mM): KCl 20, NaCl 10, $MgCl_2$ 2, $CaCl_2$ 0.03, potassium L-glutamate 120, EGTA 0.1, HEPES 5 and MgATP 5 (pH adjusted to 7.2 at 37°C with NaOH) supplemented with 2 μM Fura-2 AM (Fura-2, Molecular Probes, Eugene, Oregon). Calcium transients were measured in extracellular modified Tyrode's solution containing (in mM): NaCl 140, KCl 5.4, $CaCl_2$ 1, $MgCl_2$ 0.5, HEPES 5.5 and glucose 11 (pH adjusted to 7.6 with NaOH), supplemented with 2 μM Fura-2. Once the whole-cell configuration was achieved, myocytes were stimulated in current clamp mode at 1 Hz with a 500 ms suprathreshold stimuli. Fura-2 fluorescence was recorded using stream acquisition at 10ms intervals using a Zyla 5.5 sCMOS camera attached to an inverted Nikon TE2000-U microscope (ex 340, em 510 nm). Metamorph 7.10 was used to quantify the signal by manually tracing myocytes. An equivalent region not containing cells was used as background and subtracted.

Measurement of in vitro mitochondrial membrane potential (Ψ_m) and mitochondrial flavoprotein oxidation

Fluorescence was measured on a Hamamatsu Orca ER digital camera attached to an inverted Nikon TE2000-U microscope. Fluorescent indicator 5,5',6,6' -tetrachloro-1,1',3,3'-tetraethylbenzimidazolylcarbocyanine iodide was used to measure Ψ_m in cardiac myocytes as previously described (JC-1, 200 nM, ex = 480 nm, em = 580/535 nm, interval = 2 min, exposure =

50 ms, Molecular Probes, Eugene, Oregon) (5). Cells were incubated in calcium-free HBS (supplemented with 3 mM EGTA and 200 nM JC-1) for at least 3 h prior to measuring changes in Ψ_m. Sodium cyanide (NaCN, 40 mM, mitochondrial electron transport blocker) was added at the end of each experiment to collapse Ψ_m, confirming that the JC-1 signal was indicative of Ψ_m. Additionally, individual 580 nm and 535 nm wavelength signals were assessed in each experiment to determine whether the fluorescent indicator was accurately measuring Ψ_m. Autofluorescence was used to measure flavoprotein oxidation in cardiac myocytes based on previously described methods (ex = 480 nm, em = 535 nm, interval = 1 min, exposure = 1.5 seconds) (4, 6, 7). Carbonyl cyanide-4-(trifluoromethoxy)phenylhydrazone (FCCP, 50 μM, mitochondrial electron transport chain uncoupler) was added at the end of each experiment to increase flavoprotein oxidation confirming signal was mitochondrial in origin. Ratiometric JC-1 or flavoprotein fluorescent signals were quantified by manually tracing myocytes and subtracting an equivalent background region that did not contain cells using Metamorph 7.10. Responses to drugs were reported as a percentage increase from the basal average.

Assessment of in vivo cardiac uptake and bio-distribution of AID-TAT peptide

8-week old male BALB/c nude mice were used to assess *in vivo* cardiac uptake and bio-distribution of sulfo-Cyanine7-labeled AID(S)-TAT and AID-TAT (Cy7, W&J PharmaChem) using a CRi Maestro 2 multispectral imaging system (Cambridge Research and Instrumentation, Massachusetts, USA) based on previously described methods (8). All studies were performed in mice anesthetized with isoflurane (2-4%), followed by intraperitoneal injection of pentobarbitone sodium (240 mg/kg), as approved by the Animal Ethics Committee of The University of Western Australia in accordance with the *Australian Code for the Care and Use of Animals for Scientific Purposes* (NHMRC, 8th Edition, 2013). Mice were imaged in the absence of peptides to create a baseline spectral library (Manual Compute Spectra feature), then administered a single bolus dose of AID(S)-TAT-Cy7 or AID-TAT-Cy7 (10 μM [2 mg/kg]) via intraperitoneal injection. Anterior and posterior whole animal fluorescent images were acquired 20 and 40 min post-injection of peptides (t = 0), and every hour after for up to 4 h (exposure: 5000 ms; maximum sample size: 4.4' x 3.3'; binning: 4 x 4; excitation: deep red excitation filter set; emission: custom 700-900 nm with a 5 nm step size). Four h post-injection, hearts were excised and *ex vivo* cardiac images obtained. Using Maestro Version 3.0.1 (Caliper Life Sciences), Cy7 fluorescent signal was quantified by manually tracing hearts to obtain fluorescent read-outs (photons/cm^2/s), and subtracting baseline fluorescence (photons/cm^2/s). Bio-distribution at time of maximal AID-TAT-Cy7 uptake (t = 1 h) was quantified by manually tracing the heart, kidneys, liver and bladder, subtracting baseline fluorescence, and normalizing to total body fluorescence. Decay rates assessing clearance of the peptide via the kidneys and liver were assessed by calculating the exponential decrease in Cy7 signal from the liver and kidneys (minus baseline fluorescence) over time (t = 1-4 h).

MTT assay

The rate of reduction of 3-(4,5-Dimethyl-2-thiazolyl)-2,5-diphenyl-2H-tetrazolium bromide (MTT, Sigma-Aldrich, St. Louis, Missouri) to formazan by the mitochondrial electron transport chain was measured in intact mouse cardiac myocytes as previously described (1, 9). This reaction is dependent upon the presence of reduced nicotinamide adenine dinucleotide (NADH) and reduced nicotinamide adenine dinucleotide phosphate (NADPH), and intact mitochondrial electron transport (10). Cardiac myocytes were treated with relevant drugs in 96 well plates. MTT was added to each well to a final concentration of 0.5 mg/ml and the rate of the increase in absorbance immediately measured using a spectrophotometer at 37°C (PowerWave XS, 570 nm, reference wavelength of 620 nm). The rate of the increase in absorbance in response to treatments was expressed as a percentage of the rate of the increase prior to the addition of treatments. Each *n* represents number of replicates for each treatment group from cardiac myocytes isolated from a total of 6 *wt* (AID[S]-TAT), 4 *cTnI-G203S* (AID[S]-TAT), and 3 (AID-TAT) mice.

Immunoblot of $I_{Ca\text{-}L}$ protein

Immunoblot analysis of $I_{Ca\text{-}L}$ protein ($Ca_V1.2$) expression was performed on total heart homogenate pooled from groups of 4 *wt* or *cTnI-G203S* mice treated with AID(S)-TAT or AID-TAT (10 µM, 3x/wk/5wk). Whole heart homogenates were sonicated in 1:4 RIPA buffer, containing (in mM): NaCl 150, Tris 50, $Na_4P_2O_7$ 20, Na_3VO_4 2, NaF 1, 0.5 % Na deoxycholate, 1 % Triton X-100, 0.1 % SDS, cOmplete™ Mini EDTA-free Protease Inhibitor Cocktail tablet (Roche, 11836170001), Phosphatase Inhibitor Cocktail 1 (Sigma, P2850), pH 7.4. Total heart homogenate was centrifuged at 10,000 g for 5 minutes at 4°C. Pooled tissue homogenate protein concentration was quantified using the Bradford protein assay using BSA as a standard. 75 mg of total heart homogenate was loaded onto precast 10 % Mini-PROTEAN® TGX Stain-Free™ SDS-polyacrylamide gel (Bio-Rad Laboratories), then electrophoretically transferred to 0.2 µm PVDF membrane using a Trans-Blot® TurboTM Transfer System (Bio-Rad Laboratories). After blocking with 5% BSA in TBST, blots were probed with rabbit polyclonal anti-$Ca_V1.2$ (Alomone Labs, ACC-003, 1:200) and rabbit monoclonal anti-VDAC (porin) (Cell Signalling, #4661, 1:1000) primary antibodies. Blots were then probed with goat anti-mouse IgG H&L (HRP) preadsorbed secondary antibody (abcam, AB97040, 1:10000). Quantitative densitometry was performed on images captured using a Chemidoc imaging system (Bio-rad), using ImageJ software. Data are presented as optical density of $Ca_V1.2$ expression, normalized to loading control (porin).

Metabolomic analysis of whole heart tissue

Extraction of heart tissue: 20-week-old *wt* or *cTnI-G203S* mice (pre-cardiomyopathic) were treated with 10 µM AID(S)-TAT or AID-TAT, 3x/wk/5wk (4 animals per treatment group). Following completion of treatment regimen, mice were anaesthetized, hearts extracted and immediately snap frozen in liquid nitrogen.

Reagents: Analytical standards, 3-azido-3′-deoxythymidine (AZT), tributylamine and acetic acid were obtained from Sigma-Aldrich Pty Ltd (Castle Hill, Australia). LC-MS-grade water, acetonitrile, chloroform and methanol were obtained from Merck. Labelled standards $^{13}C_6$-D-glucose-6-phosphate and $^{13}C_5$-α-ketoglutaric acid were purchased from Novachem (Heidelberg West, Australia). Labelled L-methionine-(methyl-^{13}C, d3) and uridine-$^{13}C_9$,$^{15}N_2$-5'-monophosphate sodium salt solution were purchased from Sigma-Aldrich Pty Ltd (Castle Hill, Australia). Labelled L-phenylalanine-d7 was obtained from Sapphire Bioscience Pty Ltd (Redfern, Australia).

Metabolite extraction: Metabolites were extracted using a chloroform:methanol:water-based modified Bligh-Dyer extraction procedure (11) as follows: A bulk extraction solution (ExSol) of (*v/v/v*) 1:3:1 chloroform: methanol: water with 20 µM internal standards was made up with sufficient volume for all extractions. At all stages of the extraction process the samples and solutions were kept on ice as much as practicable.

Heart weight was recorded at the time of explantation and this was used to adjust for the volumes of ExSol and other solvents used during the tissue processing (heart weights: mean = 120 mg; SD = 14.8; min = 100 mg; max = 155 mg). A ten-fold volume of ExSol was used i.e. 500 µL per 50 mg tissue. Intact whole hearts for each mouse were placed in 2 mL Lysing Matrix D tubes (MP Biomedicals Australasia Pty Ltd, Seven Hills, Australia) with the appropriate volume of ExSol. Homogenization was achieved using a Precellys 24 tissue homogenizer fitted with a Cryolys cooling unit (Bertin Instruments, Montigny-le-Bretonneux, France), using the following settings: 6000 RPM, 3 x 30 second pulses, 45 second inter-pulse pause. Post-homogenization the tubes were centrifuged for 10 min at 10000 x g at 4°C in a Labnet Prism refrigerated microcentrifuge (Sigma-Aldrich Pty Ltd, Castle Hill, Australia). The supernatant was removed to a new vial, its volume recorded and 40 µL water/100 µL supernatant added. The sample was then vortex mixed for 10 seconds, incubated on ice for 20 min and again centrifuged for 10 mins at 10000 x g at 4°C. The aqueous supernatant was removed to a new vial, its volume recorded and 40 µL chloroform/100 µL supernatant added to wash the aqueous extract and remove lipid interferences. The sample was again vortex mixed for 10 seconds, incubated on ice for 20 min and centrifuged for 10 mins at 10000 x g at 4°C. The upper aqueous phase was removed to a new vial and its volume recorded. The aqueous extract was dried down using a Concentrator plus vacuum

centrifuge (Eppendorf, Macquarie Park, Australia), employing the V-HV program with no heating (room temperature 22 ± 1°C) and reconstituted in 95:5 water:acetonitrile as per supernatant volume. The homogenate and organic extracts/residue were combined for storage at -70°C for further extraction or protein work if necessary. Extraction blank samples were also prepared to determine solvent and reagent background. Quality control samples (QCs) were prepared by pooling aliquots of each sample (10 µL) following the recommendation by Sangster et al. (2006) (12). Final sample extracts, QCs and blanks were dispensed to glass HPLC vials with glass inserts prior to analysis.

Targeted LC-MS/MS analysis: A targeted metabolite analysis of 226 central carbon metabolites and internal standards in aqueous extracts was performed using the Agilent Metabolomics Dynamic MRM Database and Method. System suitability was checked and assessed by the acquisition of a 14 analyte panel of authentic metabolite standards. Data were acquired using an Agilent 1290 Infinity II UHPLC system (with quaternary pump) connected to an Agilent 6470 triple quadrupole mass spectrometer without split. Separation of analytes was achieved using an Agilent Zorbax RRHD Extend-C18 column (2.1 x 150 mm, 1.8 µm) fitted with an Agilent Zorbax Extend-C18 UHPLC guard column (2.1 x 5 mm, 1.8 µm) by stepped gradient elution using mobile phase A (3% methanol in water (*v/v*) containing 10 mM tributylamine and 15 mM acetic acid) and mobile phase B (97% methanol in water (*v/v*) containing 10 mM tributylamine and 15 mM acetic acid). The flow rate was set at 250 µL/min and the gradient consisted of 0-20% mobile phase B from 0 to 7.5 min, 20–45% from 7.5 to 13 min, followed by an increase to 99% for 7 min where it was then held for 4 min. The column was then "regenerated" by reverse flow of 100% acetonitrile through the analytical column and guard at 250 µL/min for 3 min, increased to 800 µL/min for 2 min, then reduced to 600 µL/min for 45 s. Thereafter the column was returned to initial conditions and was re-equilibrated with mobile phase A at 250 µL/min for 8.5 min. The column temperature was maintained at 35°C, the autosampler at 5°C and an injection volume of 5 µL was used for all injections onto the column.

The mass spectrometer was operated in negative electrospray ionization mode, and the acquisition parameters employed were as follows: capillary and nozzle voltage of -2.0 kV and -500 V respectively; sheath gas (nitrogen) 12 L/min, heated to 325°C; source gas temperature 150°C, flow 13 L/min; nebulizer pressure 45 p.s.i. Selective detection of 226 central carbon metabolites and the internal standards was achieved using dynamic multiple reaction monitoring (dMRM). Total chromatographic run time was 43 min per sample injection, with a mass spectrometric acquisition time of 25 min (prior to column regeneration and equilibration – data not acquired). After acquiring a test QC sample to assess signal abundance and the analyte dynamic range of the average sample, a ten-fold dilution set of samples was prepared and also acquired to ensure the detection of the highly abundant analytes. Where a robust signal was obtained in the dilution set this value was used for subsequent data analysis.

Data were automatically processed via a validated Agilent method, and the peak shape and area for each metabolite were visually/manually assessed, using MassHunter Quantitative Analysis v. B.08.00 software (Agilent). Processed data were curated and analysed using Excel 2016 (Microsoft Corporation, USA), SIMCA v15 (Sartorius Stedim Data Analytics AB, Umeå, Sweden), GraphPad Prism version 8.2.1 for Windows (GraphPad Software, San Diego, USA) and MetaboAnalyst v4.0.(13) Excel was used to curate the data by assessing the repeatability of the QC measurements and removing any analytes that did not pass a CV% of 25 or had mean abundance peak areas of < 600. Metabolite intensities were subsequently normalized to internal standard concentrations. SIMCA was used to assess the data for outliers and any group-related structure in the mean-centred unit variance-scaled data using principal components analysis (PCA) initially, as well as orthogonal projection to latent structures-discriminant analysis (OPLS-DA) for class-based separation where appropriate. MetaboAnalyst was used to perform one-way ANOVA on median-normalized, log-transformed data, and metabolite set enrichment analysis (MSEA) (14) was performed on those analytes deemed significant between comparator groups. Significance was corrected using the Benjamini-Hochberg method (15) within MetaboAnalyst.

Echocardiography

Echocardiographic measurement of left ventricular function were performed on mice under light methoxyflurane anesthesia using an i13L probe on a Vivid 7 Dimension ultrasound system (GE Healthcare, Little Chalfont, United Kingdom) as previously described (4, 7). Echocardiographic measurements were taken on M-mode in triplicate from separate mice at a sweep speed of 200 mm/s. Each *n* represents the average of quantitative measurements from *wt* or *cTnI-G203S* mice for each treatment group. Measurements of left ventricular end diastolic diameter (LVEDD), left ventricular end systolic diameter (LVESD), fractional shortening (FS), left ventricular posterior wall in diastole (LVDPW), left ventricular posterior wall in systole (LVSPW), interventricular septum in diastole (IVSD), interventricular septum in systole (IVSS) and end diastolic diameter (EDD) were made. FS was calculated by the formula [(LVEDD-LVESD)/EDD] x 100.

Cell size

Cell size was determined as previously described (16). Images of myocytes were captured using a Hamamatsu Orca ER digital camera attached to an inverted Nikon TE2000-U microscope. Image J was used to measure myocyte size by manually tracing myocyte images and expressing cell area in pixels (Microsoft Java 1.1.4, National Institutes for Health).

Sample preparation for confocal imaging

Following completion of treatment regimens, myocytes were isolated and prepared for confocal imaging based on previously described methods (7). Myocytes in suspension (1 mM calcium containing HBS) were plated onto laminin (20 µg/mL) coated coverslips, incubated at 37°C for 30 min, then washed twice with phosphate buffered saline (PBS), containing in mM: 13 KCl, 7.35 KH_2PO_4, 0.69 NaCl, 40.4 and 40.4 $Na_2HPO_4.7H_2O$ (pH = 7.4). Myocytes were fixed with 4% paraformaldehyde (PFA, in PBS) for 15 min at 37°C, then incubated in permeabilization buffer (0.3% Triton X-100 in PBS) for a further 15 min (37°C). Following permeabilization, myocytes were incubated in blocking buffer (containing 5% goat serum and 5% horse serum in PBS) for 1 h (37°C). Myocytes were then incubated with F-actin probe phalloidin (1:400 in 2% BSA [in PBS], Alexa Fluor® 594, ThermoFisher Scientific) for 1 h (37°C), followed by nuclear probe DAPI (1:500 in PBS, ThermoFisher Scientific) for 10 min at room temperature. Myocytes were mounted and imaged on an Olympus IX71 inverted fluorescent microscope.

Toxicity parameters

To measure kidney toxicity, urea and creatinine concentration was assessed using QuantiChrom Urea and Creatinine assay kits respectively (BioAssay Systems, Hayward CA). To measure liver toxicity, alanine transaminase (ALT) and aspartate transaminase (AST) concentration was measured using Alanine Transaminase and Aspartate Transaminase assay kits respectively (BioAssay Systems, Hayward CA). All assays were performed as per manufacturer's instructions, using a spectrophotometer (PowerWave XS).

Supporting Figures

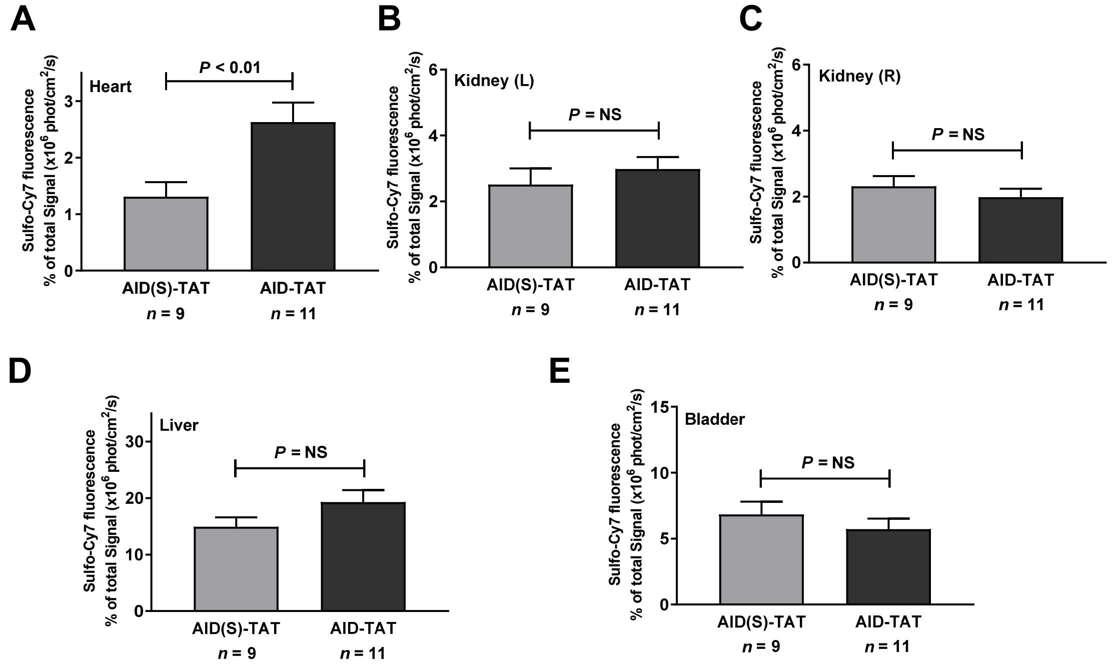

Figure S1. AID-TAT peptide targets the heart when administered *in vivo*. Mean ± SEM of Cy7 fluorescence assessed in the heart (**A**), left kidney (**B**), right kidney (**C**), liver (**D**) and bladder (**E**) for all mice (*n*) administered a single 10 µM bolus dose of AID(S)-TAT-Cy7 or AID-TAT-Cy7, 1 h post-injection. Statistical significance determined by Mann–Whitney U tests (*A*, *B*, and *D*) or unpaired *t* tests with Welch's correction (*C* and *E*). NS = not significant.

A **B**

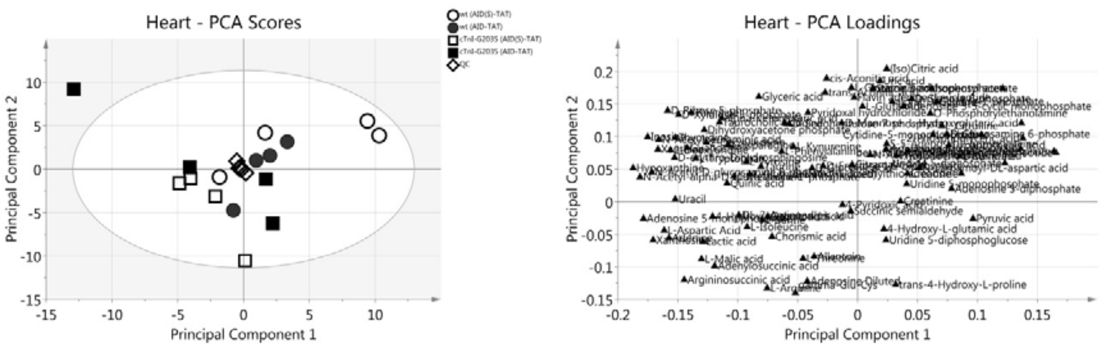

Figure S2. Principal component analysis of LC-MS/MS data acquired from heart sample extracts across all treatments. PCA-related scores (**A**, observations) and loadings (**B**, variables) plots, with the QC samples (diamonds) located centrally at the origin of the two component dimensions, confirming the success of the analytical acquisition for both sample types. Data are mean-centred, and scaled to unit variance.

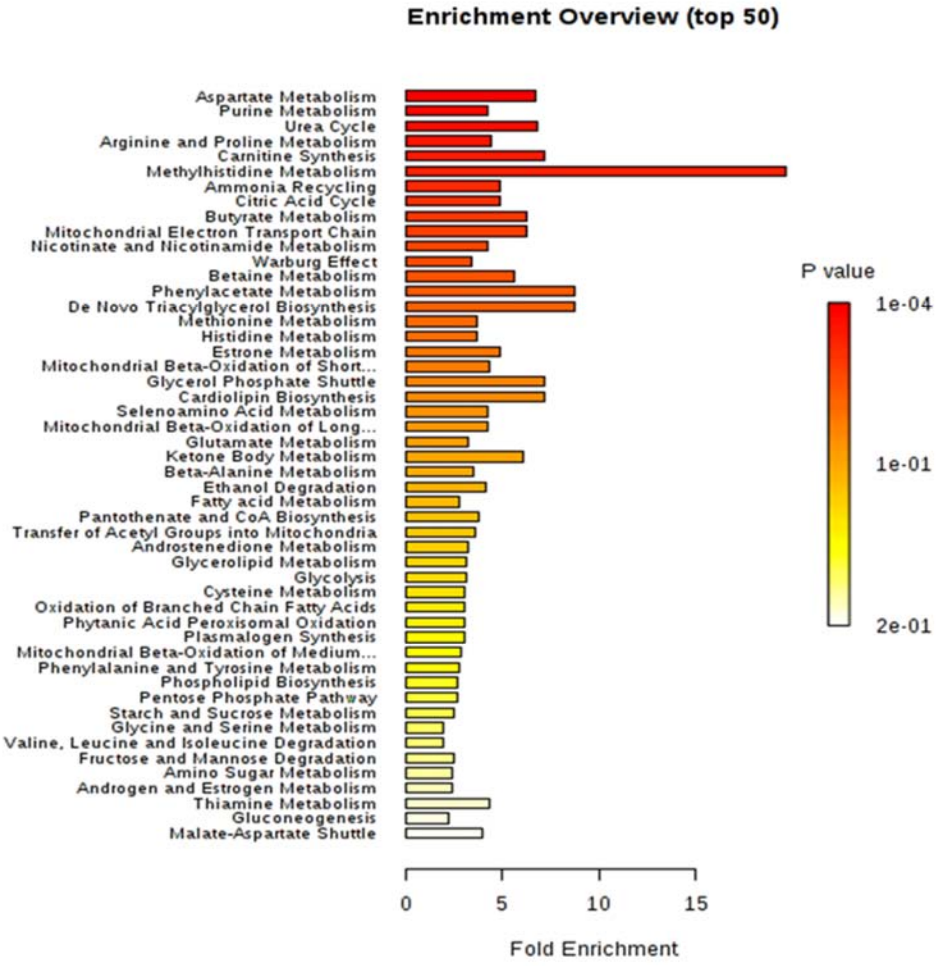

Figure S3. MSEA showing the main metabolic pathways associated with the differences observed between *wt* and *cTnI-G203S* murine heart extracts across all treatments.

Supporting Tables

Table S1. Echocardiographic parameters of mice prior to treatment with AID(S)-TAT or AID-TAT.

LVEDD (mm)	LVESD (mm)	FS (%)	LVDPW (mm)	LVSPW (mm)	IVSD (mm)	IVSS (mm)	HR (bpm)
**20 week old *wt* (*n* = 4)**							
3.61 ± 0.06	2.26 ± 0.03	36.55 ± 0.47	1.24 ± 0.08	1.45 ± 0.05	0.73 ± 0.01	0.88 ± 0.01	455 ± 10
**20 week old *cTnI-G203S* (*n* = 6)**							
3.57 ± 0.02	2.21 ± 0.01	37.92 ± 0.5	1.24 ± 0.02	1.43 ± 0.02	0.72 ± 0.01	0.87 ± 0.01	453 ± 7
**30 week old *cTnI-G203S* (*n* = 5)**							
2.99* ± 0.02	1.56* ± 0.05	47.14* ± 1.32	1.49* ± 0.05	1.59* ± 0.05	0.90* ± 0.01	1.01* ± 0.02	468 ± 16

LVEDD, left ventricular end diastolic diameter; LVESD, left ventricular end systolic diameter; FS, fractional shortening; LVDPW, left ventricular posterior wall in diastole; LVSPW, left ventricular posterior wall in systole; IVSD, interventricular septum in diastole; IVSS, interventricular septum in systole. Values reported as mean ± SEM; *$P < 0.05$ compared to 20-week-old *cTnI-G203S* mice as determined by Kruskal-Wallis tests.

Table S2. Biochemical intermediates found to be significantly different between heart extracts from *wt* or *cTnI-G203S* mice treated with AID(S)-TAT or AID-TAT.

Biochemical Intermediate	T statistic	p-value	$-\log_{10}(p)$	FDR
**L-Citrulline**	**-6.8053**	**8.52E-06**	**5.0694**	**0.00083535**
**L-Histidine**	**-5.2165**	**0.00013059**	**3.8841**	**0.0063987**
**(Iso)Citric acid**	**-4.6407**	**0.00038179**	**3.4182**	**0.012472**
**Inosine 5-monophosphate**	**-4.0964**	**0.0010898**	**2.9626**	**0.026701**
**Isopentyl acetate**	**-3.837**	**0.0018134**	**2.7415**	**0.030296**
**L-Carnitine**	**-3.7597**	**0.0021125**	**2.6752**	**0.030296**
**Pyridoxal**	**-3.714**	**0.0023122**	**2.636**	**0.030296**
**β-NAD**	**-3.6387**	**0.0026845**	**2.5711**	**0.030296**
**cis-Aconitic acid**	**-3.5991**	**0.0029038**	**2.537**	**0.030296**
**Adenylosuccinic acid**	**3.55**	**0.0032009**	**2.4947**	**0.030296**
**D-Pantothenic acid**	**-3.5091**	**0.0034721**	**2.4594**	**0.030296**
**Adenosine 5-monophosphate**	**3.4758**	**0.0037098**	**2.4307**	**0.030296**
**L-Hydroxyglutaric acid**	**-3.2959**	**0.005305**	**2.2753**	**0.039991**
**L-Glutamine**	**-3.1742**	**0.0067581**	**2.1702**	**0.047307**
S-5-Adenosyl-L-homocysteine	-3.0936	0.007932	2.1006	0.051823
Succinic acid	-2.9994	0.0095632	2.0194	0.058575
Uric acid	-2.9198	0.011196	1.9509	0.064541
L-Phenylalanine	-2.7976	0.014246	1.8463	0.077563
Adenine	2.5626	0.022559	1.6467	0.11635
Galactonic acid	2.463	0.027349	1.5631	0.12807
Adenosine	2.4612	0.027444	1.5616	0.12807
Dihydroxyacetone phosphate	2.435	0.028866	1.5396	0.12858
Uridine 5-diphosphate	-2.3641	0.033063	1.4807	0.14088
Argininosuccinic acid	2.3308	0.035226	1.4531	0.14384
Quinic acid	2.2709	0.039468	1.4038	0.15471
2-Phosphoglyceric acid	-2.2067	0.044538	1.3513	0.16788

Biochemical intermediates reported are significantly different between heart extracts from *wt* or *cTnI-G203S* at $P < 0.05$ by Student's t-test. FDR, false discovery rate (in bold font if FDR-corrected $P < 0.05$); β -NAD, β -Nicotinamide adenine dinucleotide; (Iso)Citric acid is denoted as both isomers as they are not chromatographically resolved in this analysis.

Table S3. Biochemical intermediates found to be significantly different in a four-group analysis between heart extracts from *wt* or *cTnI-G203S* mice treated with AID(S)-TAT or AID-TAT.

Biochemical Intermediate	F statistic	p-value	-$\log_{10}$(p)	FDR
**L-Citrulline**	**19.937**	**5.91E-05**	**4.2284**	**0.0057924**
**L-Histidine**	**10.086**	**0.0013373**	**2.8738**	**0.048237**
**(Iso)Citric acid**	**9.8487**	**0.0014766**	**2.8307**	**0.048237**
Isopentyl acetate	7.2561	0.0049225	2.3078	0.10301
L-Carnitine	6.9857	0.0056649	2.2468	0.10301
Inosine 5-monophosphate	6.6181	0.0068931	2.1616	0.10301
β-NAD	6.4984	0.0073579	2.1332	0.10301
Adenylosuccinic acid	5.3208	0.014553	1.8371	0.17827
cis-Aconitic acid	5.1284	0.016391	1.7854	0.17848
L-Hydroxyglutaric acid	4.5881	0.023179	1.6349	0.22077
Pyridoxal	4.4727	0.025024	1.6016	0.22077
L-Glutamine	4.3578	0.027033	1.5681	0.22077
Cytidine-5-monophosphate	4.1891	0.030328	1.5182	0.22862
Adenosine 5-monophosphate	3.8429	0.038665	1.4127	0.25495
Quinic acid	3.83	0.039022	1.4087	0.25495
D-Pantothenic acid	3.6843	0.04335	1.363	0.25875
O-Phosphorylethanolamine	3.6366	0.044885	1.3479	0.25875

Biochemical intermediates reported are significantly different between heart extracts from *wt* or *cTnI-G203S* at $P < 0.05$ by one-way ANOVA. FDR, false discovery rate (in bold font if FDR-corrected $P < 0.05$); β-NAD, β-Nicotinamide adenine dinucleotide; (Iso)Citric acid is denoted as both isomers as they are not chromatographically resolved in this analysis

Table S4. MSEA highlighting the metabolites present and influential in the enrichment of metabolic pathways associated with the differences observed between *wt* and *cTnI-G203S* murine heart extracts across all treatments.

Pathway	Total	Expected	Hits	Raw p	Holm p	FDR	Metabolites Present
**Aspartate Metabolism**	**35**	**0.889**	**6**	**0.000144**	**0.0141**	**0.0136**	**AMP; Argininosuccinic acid; IMP; Adenylsuccinic acid; L-Glutamine; Citrulline**
**Purine Metabolism**	**74**	**1.88**	**8**	**0.000277**	**0.0268**	**0.0136**	**Adenine; AMP; Adenosine; IMP; Uric acid; Adenylsuccinic acid; L-Glutamine; β-NAD**
**Urea Cycle**	**29**	**0.736**	**5**	**0.000554**	**0.0532**	**0.0181**	**AMP; Argininosuccinic acid; L-Glutamine; β-NAD; Citrulline**
**Arginine and Proline Metabolism**	**53**	**1.35**	**6**	**0.00149**	**0.142**	**0.0344**	**AMP; Argininosuccinic acid; Succinic acid; β-NAD; Citrulline; SAHc**
**Carnitine Synthesis**	**22**	**0.559**	**4**	**0.00176**	**0.165**	**0.0344**	**L-Carnitine; Succinic acid; β-NAD; SAHc**
Methylhistidine Metabolism	4	0.102	2	0.00361	0.335	0.0589	L-Histidine; SAHc
Ammonia Recycling	32	0.812	4	0.00725	0.667	0.0888	AMP; L-Histidine; L-Glutamine; β-NAD
Citric Acid Cycle	32	0.812	4	0.00725	0.667	0.0888	cis-Aconitic acid; Citric acid; Succinic acid; β-NAD

Biochemical pathways and intermediates reported are significantly different between heart extracts from *wt* or *cTnI-G203S* at *P* < 0.05 by MSEA. FDR, false discovery rate (in bold font if FDR-corrected *P* < 0.05). AMP, Adenosine monophosphate; IMP, Inosine monophosphate; SAHc, S-Adenosylhomocysteine.

Supporting References

1. H. M. Viola *et al.*, Impaired functional communication between the L-type calcium channel and mitochondria contributes to metabolic inhibition in the mdx heart. *Proceedings of the National Academy of Sciences of the United States of America* **111**, E2905-2914 (2014).
2. H. M. Viola, S. M. Davies, A. Filipovska, L. C. Hool, The L-type Ca^{2+} channel contributes to alterations in mitochondrial calcium handling in the *mdx* ventricular myocyte. *Am J Physiol Heart Circ Physiol* **304**, H767-775 (2013).
3. T. D. O'Connell, M. C. Rodrigo, P. C. Simpson, Isolation and culture of adult mouse cardiac myocytes. *Methods in molecular biology (Clifton, N.J.)* **357**, 271-296 (2007).
4. H. M. Viola *et al.*, The L-type Ca channel facilitates abnormal metabolic activity in the cTnI-G203S mouse model of hypertrophic cardiomyopathy. *J Physiol* **594**, 4051-4070 (2016).
5. H. M. Viola, P. G. Arthur, L. C. Hool, Transient Exposure to Hydrogen Peroxide Causes an Increase in Mitochondria-Derived Superoxide As a Result of Sustained Alteration in L-Type Ca2+ Channel Function in the Absence of Apoptosis in Ventricular Myocytes. *Circulation research* **100**, 1036-1044 (2007).
6. Y. Yaniv *et al.*, Ca2+-regulated-cAMP/PKA signaling in cardiac pacemaker cells links ATP supply to demand. *Journal of molecular and cellular cardiology* **51**, 740-748 (2011).
7. H. M. Viola *et al.*, The Role of the L-Type Ca2+ Channel in Altered Metabolic Activity in a Murine Model of Hypertrophic Cardiomyopathy. *J Am Coll Cardiol: Basic to Translational Science* **1**, 61-72 (2016).
8. P. S. R. Naidu *et al.*, Elucidating the Inability of Functionalized Nanoparticles to Cross the Blood-Brain Barrier and Target Specific Cells in Vivo. *ACS Appl Mater Interfaces* **11**, 22085-22095 (2019).
9. H. M. Viola, P. G. Arthur, L. C. Hool, Evidence for regulation of mitochondrial function by the L-type Ca2+ channel in ventricular myocytes. *Journal of molecular and cellular cardiology* **46**, 1016-1026 (2009).
10. M. V. Berridge, A. S. Tan, Characterization of the cellular reduction of 3-(4,5-dimethylthiazol-2-yl)-2,5-diphenyltetrazolium bromide (MTT): subcellular localization, substrate dependence, and involvement of mitochondrial electron transport in MTT reduction. *Arch Biochem Biophys* **303**, 474-482 (1993).
11. E. G. Bligh, W. J. Dyer, A rapid method of total lipid extraction and purification. *Can J Biochem Physiol* **37**, 911-917 (1959).
12. T. Sangster, H. Major, R. Plumb, A. J. Wilson, I. D. Wilson, A pragmatic and readily implemented quality control strategy for HPLC-MS and GC-MS-based metabonomic analysis. *Analyst* **131**, 1075-1078 (2006).
13. J. Chong *et al.*, MetaboAnalyst 4.0: towards more transparent and integrative metabolomics analysis. *Nucleic Acids Res* **46**, W486-W494 (2018).
14. J. Xia, D. S. Wishart, MSEA: a web-based tool to identify biologically meaningful patterns in quantitative metabolomic data. *Nucleic Acids Res* **38**, W71-77 (2010).
15. Y. Benjamini, Y. Hochberg, Controlling the False Discovery Rate: A Practical and Powerful Approach to Multiple Testing. *Journal of the Royal Statistical Society. Series B (Methodological)* **57**, 289-300 (1995).
16. V. Seenarain *et al.*, Evidence of altered guinea pig ventricular cardiomyocyte protein expression and growth in response to a 5 min in vitro exposure to H(2)O(2). *Journal of proteome research* **9**, 1985-1994 (2010).
